# Gene Editing of a Susceptibility LncRNA Enhances Broad‐Spectrum Disease Resistance in Rice without Developmental Trade‐Offs

**DOI:** 10.1002/advs.202505671

**Published:** 2025-08-18

**Authors:** Wen‐long Zhao, Ye Cheng, Jia‐hui Huang, Jun‐jie Feng, Hui‐yin Pang, Yi‐chao Qin, Zheng‐tong Chen, Yu Cheng, Jian‐ping Lian, Yan‐fei Zhou, Rui‐rui He, Meng‐qi Lei, Zi‐qin Cao, Lu Yang, Chao Yuan, Jie Jiang, Yue‐qin Chen, Yu‐chan Zhang

**Affiliations:** ^1^ Guangdong Provincial Key Laboratory of Plant Resources State Key Laboratory for Biocontrol School of Life Science Sun Yat‐Sen University Guangzhou 510275 P. R. China

**Keywords:** broad‐spectrum resistance, lncRNA, NAA10, NAA15, rice

## Abstract

The identification and genomic editing of defense‐related genes to confer resistance to pathogens is an effective and promising strategy for use in crop breeding. However, resistance is often associated with growth inhibition, a phenomenon referred to as the “trade‐off” effect, making enhancing resistance without sacrificing yield challenging. In this study, a novel strategy is presented to enhance broad‐spectrum resistance in crops without yield loss by editing susceptibility lncRNAs. *RESIS*, a pathogen‐induced lncRNA that acquired its function in the pathogen response during domestication, is identified. Upon pathogen invasion, *RESIS* is activated by effector‐binding elements on its promoter and subsequently binds to NAA15 and NAA10, two core components of the NatA complex. *RESIS* enables NAA10 to interact with NAA15 through a sequence that evolves in cultivated rice, enhancing the activity of the NatA complex in the N‐terminal acetylation of proteins. *RESIS* knockout suppresses this process and increases translation during pathogen invasion, conferring resistance to both fungal and bacterial diseases without the growth inhibition typically associated with the direct knockout of the NatA complex. These findings highlight the potential of susceptibility lncRNAs as promising target loci for improving crop broad‐spectrum disease resistance without detrimental effects on growth, offering significant prospects for practical applications.

## Graphical abstract

Editing susceptibility lncRNAs offers a strategy to enhance disease resistance without yield loss. *RESIS*, a domestication‐acquired, pathogen‐induced lncRNA, promotes protein N‐terminal acetylation through its interaction with NatA complex. Knockout of *RESIS* increases translation and confers broad‐spectrum resistance to both fungal and bacterial pathogens without causing growth inhibition, highlighting its potential as a valuable target for crop improvement.

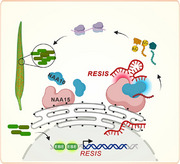

## 1 Introduction

Genomic editing holds significant promise for enhancing quantitative traits and is regarded as one of the most effective strategies for crop improvement.^[^ ^1^, ^2^ ^]^ However, the “trade‐off” effect between growth and development often complicates the application of genetic modifications to resistance genes, as interventions targeting resistance typically result in a reduction in yield traits, and vice versa.^[^ ^3^, ^4^, ^5^, ^6^, ^7^ ^]^ Rice has undergone thousands of years of domestication, transitioning from wild rice to cultivated rice. Compared with its wild ancestor, cultivated rice has shown considerable improvements in yield traits, albeit at the expense of reduced resistance. As a consequence, rice crops are susceptible to a variety of diseases, which negatively impact both yield and quality, leading to significant economic losses.^[^ ^8^, ^9^ ^]^ For example, bacterial blight (BB), caused by *Xanthomonas oryzae* pv. *oryzae* (*Xoo*), and rice blast, caused by *Magnaporthe oryzae* (*M*. *oryzae*), are among the most widespread and destructive rice diseases globally and are classified among the top ten most critical bacterial and fungal plant diseases.^[^ ^10^ ^]^

Understanding the molecular mechanisms underlying pathogen infection, as well as identifying the factors that determine host susceptibility or resistance, are key strategies for enhancing plant resistance.^[^ ^11^ ^]^ During the evolutionary arms race between plants and pathogenic microorganisms, plant defense mechanisms evolved into complex interactions between resistance (*R*) genes and susceptibility (*S*) genes.^[^ ^11^, ^12^ ^]^ *S* genes contribute to plant susceptibility to a pathogen, and the knockout of *S* genes can enhance resistance. However, *S* genes are also essential for plant growth, making their knockout for increasing resistance impractical in crop breeding because of the associated yield loss.^[^ ^13^, ^14^, ^15^, ^16^ ^]^ An alternative approach is to introduce mutations in the effector‐binding elements (EBEs) in the promoters of *S* genes. These EBEs serve as binding sites for transcriptional activator‐like (TAL) effectors secreted by pathogens to activate *S* gene expression.^[^ ^17^ ^]^ For example, TAL effectors bind to EBEs in the promoters of *SWEET* genes. CRISPR/Cas9‐mediated genome editing or TALEN‐based gene editing of the EBEs in the promoters of *SWEET* genes endows rice lines with robust, broad‐spectrum resistance without yield reduction.^[^ ^18^, ^19^, ^20^, ^21^, ^22^ ^]^ However, not all *S* genes contain suitable target sites for gene editing to enhance resistance. Therefore, it is crucial to identify alternative strategies or *S* genes that do not affect plant growth.

Long non‐coding RNAs (lncRNAs) are a class of molecules that are highly abundant and rapidly evolve and whose functions are not well defined.^[^ ^23^, ^24^ ^]^ One prominent feature of lncRNAs is their sensitivity to environmental changes, with various lncRNAs displaying unique expression profiles in response to different environmental stimuli.^[^ ^25^, ^26^, ^27^, ^28^ ^]^ This sensitivity is particularly pronounced in plants, which, owing to their inability to move, require more robust environmental response mechanisms. Transcriptome data consistently show that lncRNAs are among the most dynamically regulated molecules. Some stress‐induced lncRNAs are expressed only under specific stimuli.^[^ ^25^, ^27^, ^29^, ^30^ ^]^ While the functions of these inducible lncRNAs are not yet fully understood, it is hypothesized that some lncRNAs function as susceptibility lncRNAs (*S* lncRNAs) and could present fewer developmental defects when targeted for loss‐of‐function mutations.

To validate this hypothesis and identify *S* lncRNAs involved in the pathogen response, we screened for conserved, pathogen‐induced lncRNAs and identified one, *RESIS*, that functions as an *S* lncRNA during pathogen infection. *RESIS* knockout significantly enhanced broad‐spectrum resistance to both fungal and bacterial diseases without causing any developmental trade‐offs. *RESIS* was found to interact with the NatA complex, a key complex that acetylates ≈40% of the proteomes in both animals and plants, regulating protein fate in eukaryotes.^[^ ^31^, ^32^, ^33^ ^]^ Moreover, *RESIS* is required for the interaction between NAA15 and NAA10, two core members of the NatA complex. Importantly, *RESIS* evolved a specific domain during domestication that guides NAA10 to bind to NAA15 and catalyzes the N‐terminal acetylation of proteins involved in ribosome assembly and translation pathways. Our findings demonstrate that *S* lncRNAs can serve as novel target loci for enhancing crop resistance without compromising growth, indicating substantial potential for practical applications.

## 2 Results

### 2.1 The Expression of a Set of Conserved lncRNAs was Induced by Rice Pathogen Invasion

**Figure 1:**
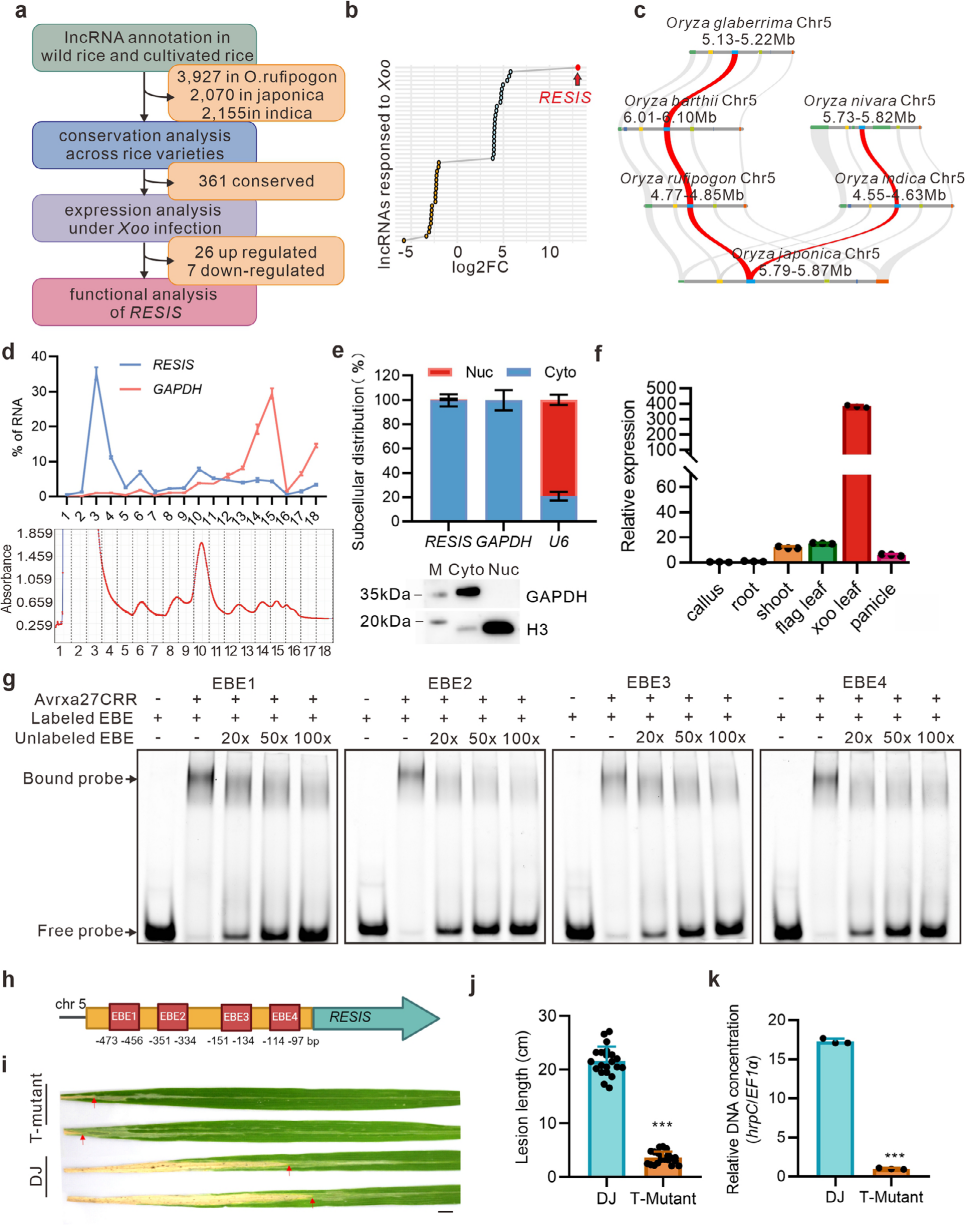
Identification of pathogen‐induced lncRNAs and initial functional characterization of *RESIS*. a) Schematic representation of the identification of conserved, pathogen‐induced lncRNAs. b) Volcano plot illustrating the variation ratios for pathogen‐induced lncRNAs in response to *Xoo* infection, with *RESIS* highlighted as the most responsive lncRNA. c) Local collinearity showing the conservation of *RESIS* from wild rice (*O*. *glaberrima*, *O*. *barthii*, *O*. *rufipogon*, and *O*. *nivara*) to cultivated rice (*O*. *indica* and *O*. *japonica*). *RESIS* is indicated in red. d) Polysome profiling was used to assess the translational potential of *RESIS*, with *GAPDH* used as the control. e) Nuclear–cytoplasmic fractionation. Western blot analysis of GAPDH and H3 confirmed the fractionation efficiency. Cyto, cytoplasm; Nuc, nucleus. f) The relative expression patterns of *RESIS* in five tissues and flag leaf after *Xoo* infection were analyzed; roots were used as a control. g) Electrophoretic mobility shift assay (EMSA) showing the specific binding of the Avrxa27 central repeat region (Avrxa27CRR) to the EBE regions in the *RESIS* promoter. h) Schematic diagram of the EBEs recognized by AvrXa27 in the *RESIS* promoter. i) Leaves of wild‐type control DJ and the *RESIS* T‐DNA mutant plants inoculated with *Xoo* strain PXO99A. Photographs were taken 14 days after inoculation. Scale bar, 1 cm. j) Lesion lengths of DJ and *RESIS* T‐mutant plants at 14 days after inoculation with the *Xoo* strain PXO99A. k) qPCR analysis of the relative *Xoo* DNA concentration between bacterial *hrpC* and rice *EF1α* in DJ and *RESIS* T‐mutant plants. Statistics: n = 3 per group for d–f,k); n = 30 per group for j. Data shown represent the mean ± SD. Unpaired, two‐tailed *t*‐tests were performed. ^***^ *p* <0.001.

During rice domestication, most lncRNAs underwent rapid evolution with low conservation. However, a small number of lncRNAs remain highly conserved, suggesting that they may have important and stable functions, although most of their functions remain unclear.^[^ ^34^ ^]^ Among these lncRNAs, some may be activated by specific stimuli. Given that wild rice has a lower yield but higher resistance than cultivated rice does, we focused on lncRNAs that function in the disease response of rice.^[^ ^35^ ^]^ We reanalyzed publicly available transcriptome deep sequencing (RNA‐seq) data from *O*. *rufipogon*, *O*. *japonica*, and *O. indica* (PRJNA437000^[^ ^36^ ^]^ and PRJNA756899^[^ ^37^ ^]^). To assess their conservation through domestication, we performed a collinearity analysis.^[^ ^38^, ^39^ ^]^ Together, these analyses yielded 361 lncRNAs showing sequence conservation and collinearity with their neighboring genes. To identify pathogen‐induced lncRNAs, strand‐specific RNA‐seq was performed using rice leaf samples infected with or without *Xanthomonas oryzae* (*Xoo*), with three biological replicates. Referencing published datasets (PRJNA544880^[^ ^40^ ^]^ and PRJNA1043097), a total of 744 *Xoo*‐induced lncRNAs were identified, which we then intersected with conserved lncRNAs. Ultimately, 33 conserved lncRNAs were differentially expressed during *Xoo* invasion, 26 of which were activated by *Xoo* infection (**Figure** **1**a,b; Figure S1a,b, and Table S1, Supporting Information).

Among the 26 lncRNAs, the lncRNA *Os05g0195000* was highly conserved in wild rice, japonica, and indica and was significantly induced by pathogen infection; thus, we named it *RESIS* (*REsponsIve to Xoo and conServed*) (Figure 1c; Figure S1c, Supporting Information). We experimentally validated its 5′ and 3′ ends through the rapid amplification of cDNA ends (RACE). *RESIS* was not associated with polysome (Figure 1d). We then predicted the open reading frames (ORFs) in the *RESIS* transcript using NCBI ORF Finder and identified four ORFs greater than 30 nt. We then fused these four ORFs into the *pRTVcHA* vector and tested their translation capacity using a western blot in rice protoplast. Two reported small peptides, OsEPFL6 and OsRALF26, were used as positive controls.^[^ ^41^, ^42^ ^]^ The results showed that none of the four ORFs exhibited translation capacity, which confirmed its lack of coding potential (Figure S1d,e, Supporting Information). After seedlings were fractionated into nuclear and cytoplasmic components, we found that *RESIS* was primarily localized in the cytoplasm (Figure 1e). Expression pattern analysis revealed that the basal expression of *RESIS* was very low but significantly increased after pathogen infection (Figure 1f). The *RESIS* promoter was predicted to contain 4 EBEs, which have been reported to recruit TAL effectors and initiate the transcription of *S* genes^[^ ^43^ ^]^ (Figure 1h; Figure S1f, Supporting Information). We have performed an Electrophoretic mobility shift assay (EMSA) using both EBEs and four control probes, which contained sequences adjacent to the EBEs with the same length. These were incubated with Avra27CRR. The gel shift results showed that the four EBEs, but not the four control probes, were bound by the Avrxa27 central repeat region (Avrxa27CRR) TAL effector, which was responsible for *Xoo* infection‐induced *RESIS* expression (Figure 1g,h; Figure S1f–i, Supporting Information).

To analyze the functions of *RESIS*, we first obtained a T‐DNA insertion mutant (*RESIS* T‐DNA) that disrupted the structure of *RESIS* and abolished *RESIS* expression (Figure S1j, Supporting Information). Wild‐type (WT) plants and *RESIS* T‐DNA plants presented similar vegetative and reproductive growth patterns (Figure S1k, Supporting Information). To investigate the potential role of *RESIS* in plant immunity, we assessed the responses of *RESIS* T‐DNA and WT plants to *Xoo*. Upon inoculation with the *Xoo* strain PXO99A, *RESIS* T‐DNA plants presented significantly higher resistance to *Xoo* than did WT plants, as evidenced by quantitative measurements of leaf lesion length and pathogen‐specific gene expression levels (Figure 1i–k). These results indicate that *RESIS* is induced by rice blast infection and may function as an *S* lncRNA.

### 2.2 *RESIS* Knockout Enhances Broad‐Spectrum Resistance in Rice without Compromising Grain Yield

**Figure 2:**
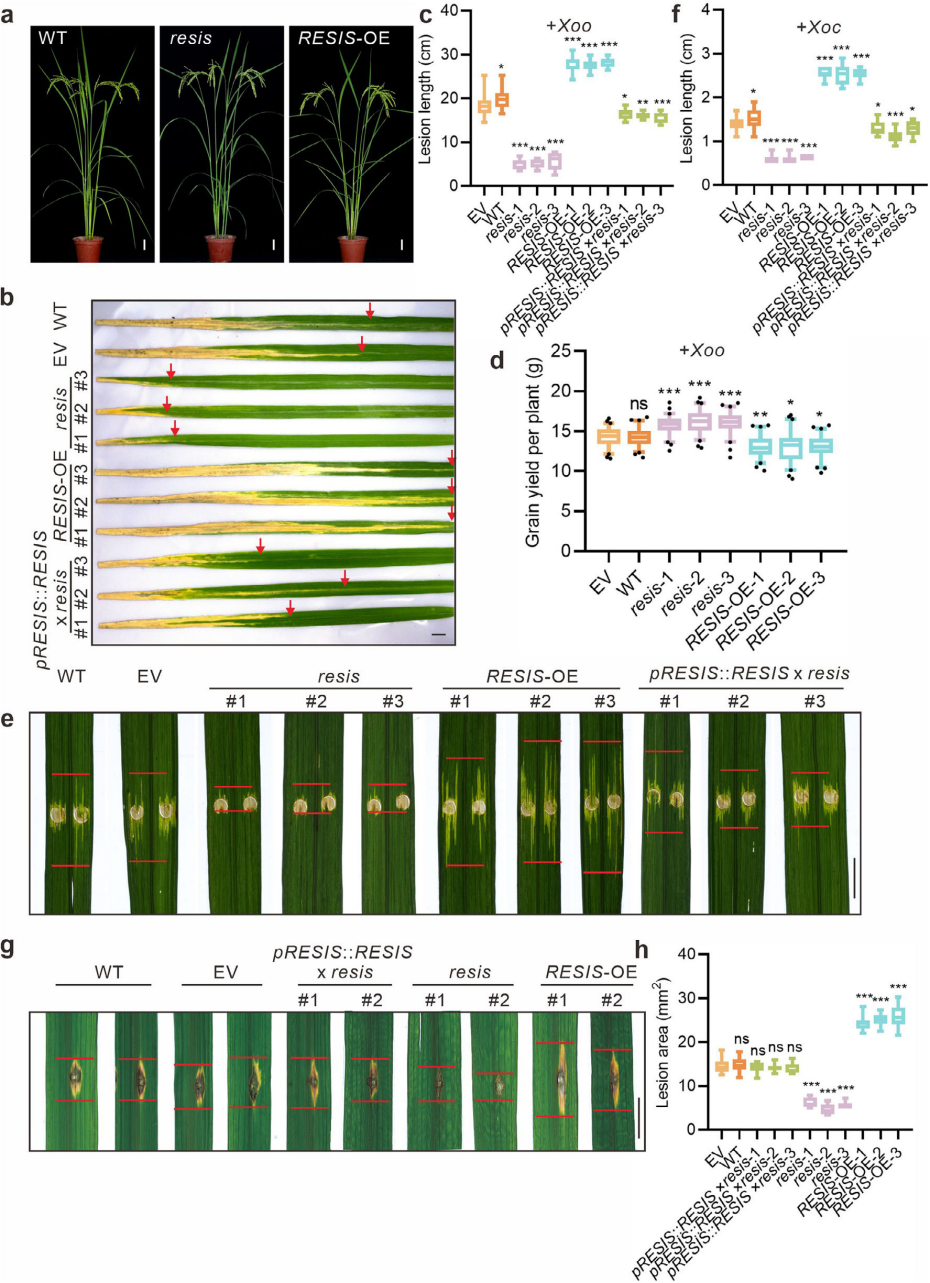
Functional analysis of *RESIS*. a) Whole WT, *resis* and *RESIS*‐OE mutant plants at the heading stage. Scale bars, 5 cm. b) Leaves of the WT, EV, *resis*, *RESIS*‐OE and *pRESIS*::*RESIS* × *resis* plants inoculated with *Xoo* strain PXO99A. Photographs were taken 14 days after inoculation. Scale bar, 1 cm. The red arrows indicate lesion length. c) The lesion length in EV, WT, *resis*, *RESIS*‐OE and *pRESIS*::*RESIS* × *resis* plants at 14 days after inoculation with *Xoo* strain PXO99A. d) Grain yield per plant for EV, WT, *resis* and *RESIS*‐OE plants after *Xoo* inoculation. e) Phenotypes of lesion expansion in WT, EV, *resis*, *RESIS*‐OE and *pRESIS*::*RESIS* × *resis* plants challenged with *Xoc* strain GDXc267. Scale bar, 1 cm. The red lines indicate lesion length. f) The lesion length in EV, *resis*, *RESIS*‐OE and *pRESIS*::*RESIS* × *resis* plants at 14 days after inoculation with *Xoc* strain GDXc267. g) Phenotypes of the leaves at tillering stage of WT, EV, *pRESIS*::*RESIS* × *resis*, *resis* and *RESIS*‐OE plants punch‐inoculated with the *M*. *oryzae* strain 08‐T19. Scale bar, 1 cm. h) The lesion area of (g) was measured at 7 dpi. Statistics: n ≥ 10 per group for c; n = 30 per group for d; n ≥ 9 per group for f; and n ≥ 6 per group for h. The data shown represent means ± SD. Unpaired, two‐tailed *t*‐tests were performed. ^*^ *p* <0.05, ^**^ *p* <0.01 and ^***^ *p* <0.001.

To further explore the function of *RESIS*, we generated *RESIS*‐knockout (*resis*) plants via CRISPR/Cas9 and *RESIS*‐overexpression lines (*RESIS*‐OE). In the *resis* lines, the promoter region was knocked out, resulting in elimination of the *RESIS* transcript (Figure S2a–c, Supporting Information). Both wild‐type (WT) plants and empty vector (EV)‐transformed plants were used as controls. Phenotypic screening revealed that the vegetative growth of all the transgenic plants was similar to that of the control plants, which was consistent with observations for the *RESIS* T‐DNA plants (**Figure** **2**a; Figure S2d, Supporting Information). We systematically analyzed grain yield‐related traits after harvest, including panicle number, panicle size, plant height, grain weight, yield per plant, and biomass, and found that the *resis* lines exhibited a modest increase in panicle size, whereas the *RESIS*‐OE lines presented no significant changes in grain yield‐related traits (Figure S2e–m, Supporting Information).

To induce rice *Xoo* infection, rice flag leaves were inoculated with *Xoo* strain PXO99A using the leaf‐clipping method. After inoculation, the plants were maintained under controlled environmental conditions conducive to pathogen proliferation for two weeks. In WT plants, the expression of *RESIS* significantly increased starting at 6 h after *Xoo* infection, indicating successful infection (Figure S3a, Supporting Information). The lengths of the leaf lesions and the pathogen load were analyzed to quantify disease severity. Notably, the leaf lesions of the *resis* plants were significantly shorter than those in the control plants (Figure 2b,c). Similarly, DNA‐based quantitative PCR (qPCR) analysis of *hrpC*, a specific *Xoo* pathogenic gene, revealed a much lower pathogen load in *resis* leaves. Conversely, *RESIS*‐OE plants were more sensitive to *Xoo* infection than control plants (Figure S3b, Supporting Information). The grain yield‐related traits of the plants infected with bacterial blight were subsequently analyzed to assess their performance under biotic stress. After *Xoo* infection, the control plants experienced ≈15.4% yield loss, including a decrease in the seed setting rate and grain weight (Figure 2d; Figure S3c,d, Supporting Information). However, compared with uninfected plants, *resis* plants maintained a normal grain yield, whereas the yield loss of the *RESIS*‐OE plants increased to ≈21.7% (Figure 2d; Figure S2k, Supporting Information). Thus, *RESIS* mitigated yield loss due to rice bacterial blight.

To verified the functions of EBEs in the resistance variation conferred by *RESIS* expression, we then transferred the *pRESIS*::*RESIS* vector, an empty vector, and a *pRESISmut*::*RESIS* vector (in which the EBEs were mutated) into *resis* protoplasts with *Xoo*. The expressions of *RESIS* and the levels of *Xoo* invasion were then examined. The results showed that *RESIS* expression was stimulated by *Xoo* infection, but this stimulation did not occur when EBEs were mutated. Consistently, *Xoo* invasion was significantly promoted by *RESIS* expression, but no significant difference in the invasion rate was observed between protoplast transferred with the empty vector or the *pRESISmut*::*RESIS* vector. This indicates the specificity and relevance of the Avra27CRR‐EBE interaction on the *RESIS* promotor (Figure S3e, Supporting Information). To confirm that the resistance to *Xoo* was due to *RESIS* knockout, we expressed *RESIS* under its native promoter in *resis* plants (*pRESIS*::*RESIS* × *resis*) and evaluated their resistance and phenotypic characteristics. The *pRESIS*::*RESIS* × *resis* plants presented similar phenotypes to those of the control plants during *Xoo* infection (Figure 2b,c; Figure S3b, Supporting Information). This restoration of the *resis* plant phenotype supports the conclusion that the observed resistance to *Xoo* was indeed a result of the absence of *RESIS* expression.

Next, we investigated whether *RESIS* regulates broad‐spectrum resistance. Plants were treated with both *Xanthomonas oryzae pv. oryzicola* (*Xoc*), which causes bacterial leaf streak, and *M*. *oryzae*, the causal pathogen of destructive blast disease. *resis* plants exhibited strong resistance to *Xoc*, whereas *RESIS*‐OE plants were more susceptible to *Xoc*. The resistance of the *pRESIS*::*RESIS* × *resis* plants was similar to that of the control plants during *Xoc* infection (Figure 2e,f; Figure S3f, Supporting Information). *RESIS* was also induced by *M*. *oryzae* starting 48 h after infection, possibly due to the presence of binding sites for pathogen‐induced TFs, such as *OsERF040*,^[^ ^44^ ^]^ *OsERF83*,^[^ ^45^ ^]^ and *OsMYB30* ^[^ ^46^ ^]^ in the promoter of *RESIS*, a site reported to be involved in responses to *M*. *oryzae* (Figure S3g,h, Supporting Information). Similarly, an analysis of lesion area and pathogen load quantification at both seedling and tillering stages, under both spray and punch inoculation methods, consistently demonstrated that the *resis* plants presented significantly higher resistance to blast disease than control plants did (Figure 2g,h; Figure S3i–n, Supporting Information). Collectively, these findings suggest that knocking out *RESIS* can enhance rice resistance to both fungal and bacterial diseases without compromising growth or yield, making it a promising target for the molecular breeding of broad‐spectrum disease resistance rice varieties.

### 2.3 *RESIS* Binds to the NatA Complex in Rice Plants

**Figure 3:**
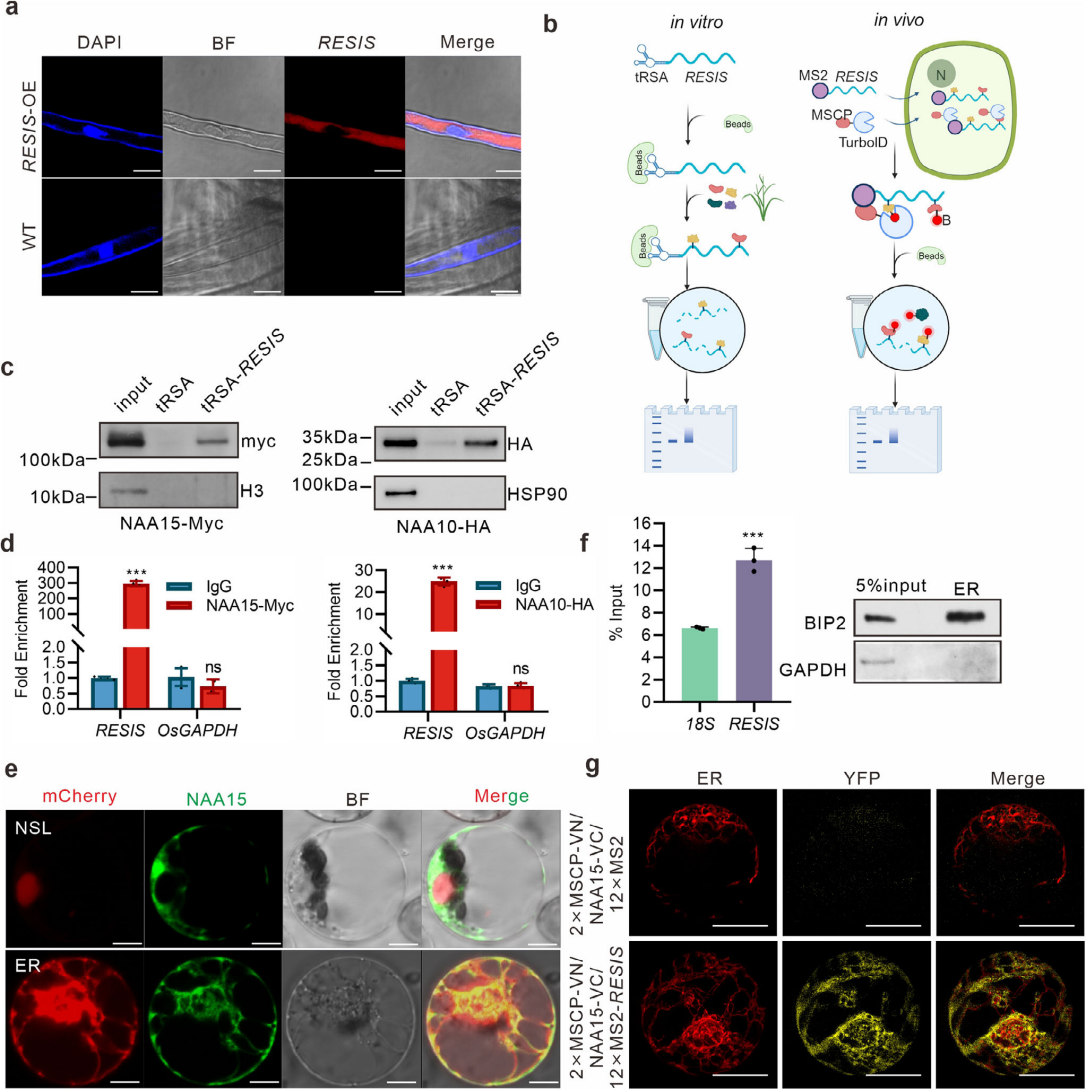
Identification of target proteins that interact with *RESIS*. a) Subcellular localization of Cy3‐labeled *RESIS* mRNA in the root hair cells of wild‐type (WT) and *RESIS*‐OE plants. Nuclei were stained with DAPI. Scale bars, 10 µm. b) Schematic diagram of the identification of *RESIS*‐interacting proteins: tRSA‐*RESIS* pull‐down assay (left) and TurboID‐mediated proximity labeling of *RESIS* (right). c) RNA pull‐down assays of *RESIS* with NAA15 and NAA10. d) RNA immunoprecipitation assays of *RESIS* with NAA15 and NAA10. e) Subcellular localization of NAA15. The fluorescence signals in rice protoplasts were detected by confocal microscopy. Red fluorescence, nuclear marker HY5‐mCherry or ER‐mCherry; green fluorescence, NAA15‐eGFP; BF, bright field. Scale bars, 10 µm. f) Isolation of the endoplasmic reticulum (ER) and enrichment analysis of *RESIS* on the ER. Left: the proportions of *RESIS* and 18S rRNA in the ER component. Right: the detection of separation effect of ER component. g) TriFC assays of *RESIS* and NAA15 in WT protoplasts were performed via structured illumination microscopy (SIM). An equal amount of 2 × MSCP‐VN, NAA15‐VC and 12 × MS2 was used as a negative control. Scale bars, 10 µm. Statistics: n = 3 per group for d,f). Data shown represent the mean ± SD. Unpaired, two‐tailed *t*‐tests were performed. ^***^ *p* <0.001.

The above results demonstrate that *RESIS* can enhance rice resistance without compromising grain yield. Next, we sought to understand how *RESIS* achieves this effect. Fractionation assays revealed that *RESIS* predominantly accumulates in the cytoplasm, a finding corroborated by RNA fluorescence in situ hybridization in rice roots overexpressing *RESIS* (**Figure** **3**a). While most lncRNAs localize in the nucleus and regulate gene expression in a *cis* or *trans* manner,^[^ ^25^ ^]^ cytoplasmic lncRNAs may interact with proteins to exert functions distinct from their nuclear counterparts. We examined whether *RESIS* affects the expression of neighboring protein‐coding genes. *RESIS* is near two protein‐coding genes (*LOC_Os05g10650* and *LOC_Os05g10660*) within a 30 kb region (Figure S4a, Supporting Information). RT–qPCR analyses of WT and transgenic plants revealed no correlation between *RESIS* and these two genes, indicating that *RESIS* does not regulate the expression of neighboring genes (Figure S4b, Supporting Information).

To identify targets of *RESIS*, we employed both RNA pull‐down and TurboID assays, followed by mass spectrometry, to identify proteins that interact with the lncRNA in vitro and in vivo, respectively (Figure 3b; Figure S4c,d, Supporting Information). Interestingly, N‐alpha‐acetyltransferase 15 (NAA15) emerged as the most abundant candidate protein in both the RNA pull‐down and TurboID assays (Tables S2 and S3, Supporting Information). Furthermore, not only NAA15 but also other members of the N‐acetyltransferase A complex (NatA) complex,^[^ ^31^, ^32^, ^33^ ^]^ specifically NAA10 and Huntingtin Interacting Protein K (HYPK), were identified as interacting partners of *RESIS* (Table S2, Supporting Information).

To validate the interaction between *RESIS* and these candidate proteins, we conducted transfer RNA (tRNA)‐scaffolded streptavidin aptamer (tRSA)‐RNA pull‐down assays^[^ ^47^, ^48^ ^]^ using in vitro–transcribed tRSA‐tagged *RESIS* (Figure S4c, Supporting Information). The assays revealed a strong interaction between *RESIS* and NAA15 and significant binding between *RESIS* and NAA10; however, HYPK did not interact with *RESIS* (Figure 3c; Figure S4e, Supporting Information). An RNA immunoprecipitation (RIP) assays using NAA15‐Myc and NAA10‐HA fusion proteins in rice protoplasts confirmed the strong interactions of both NAA15 and NAA10 with *RESIS* (Figure 3d). We also performed an in vitro RNA EMSA with *RESIS*. The result showed that *RESIS* directly binds to both NAA15 and NAA10 (Figure S4f, Supporting Information).

The NatA complex acetylates ≈40% of the human and plant proteomes in a co‐translational manner, thus regulating protein fate in eukaryotes.^[^ ^31^, ^33^ ^]^ The interaction of the NatA complex with ribosomes is mediated by NAA15.^[^ ^32^, ^33^, ^49^ ^]^ No canonical RNA binding region has been reported in the NAA15 and NAA10 proteins. However, they have been identified as RNA‐binding proteins (RBPs) in *Arabidopsis* and included in RNA‐binding protein databases.^[^ ^50^, ^51^, ^52^ ^]^ We first examined the subcellular localization of the NatA complex. The NAA15‐eGFP fusion protein partially co‐localized with an endoplasmic reticulum (ER) marker (Figure 3e). To explore the spatial overlap between *RESIS* and the NatA complex, we isolated ER fractions and measured *RESIS* abundance. The results revealed that *RESIS* is an ER‐associated lncRNA (Figure 3f). To investigate the in vivo association of *RESIS* transcripts with NatA, we employed a trimolecular fluorescence complementation (TriFC) assay using an MS2 system, the results of which demonstrated that *RESIS* interacted with NAA15 on the ER (Figure 3g). Taken together, our results demonstrate that *RESIS* binds to the NatA complex in the ER.

### 2.4 NatA Negatively Regulates Resistance and is Essential for the Normal Growth of Rice Plants

**Figure 4:**
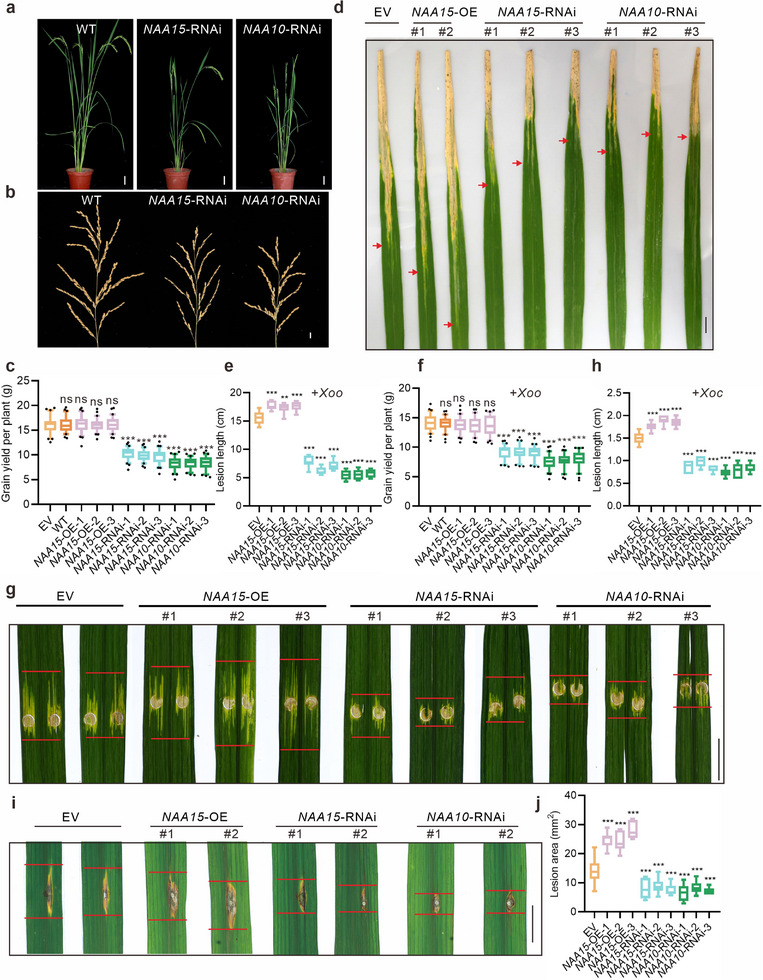
Phenotypic analysis of the NatA complex. a) Whole WT, *NAA15*‐RNAi and *NAA10*‐RNAi mutant plants at the heading stage. Scale bars, 5 cm. b) Representative panicles of WT, *NAA15*‐RNAi and *NAA10*‐RNAi plants. Scale bar, 1 cm. c) Grain yield per plant for EV, WT, *NAA15*‐OE, *NAA15*‐RNAi and *NAA10*‐RNAi plants. d) Leaves of EV, *NAA15*‐OE, *NAA15*‐RNAi and *NAA10*‐RNAi plants inoculated with the *Xoo* strain PXO99A. Photographs were taken 14 days after inoculation. Scale bar, 1 cm. The red arrows indicate lesion length. e) The lesion length in EV, *NAA15*‐OE, *NAA15*‐RNAi and *NAA10*‐RNAi plants at 14 days after inoculation with the *Xoo* strain PXO99A. f) Grain yield per plant for EV, WT, *NAA15*‐OE, *NAA15*‐RNAi and *NAA10*‐RNAi plants after *Xoo* inoculation. g) Phenotypes of lesion expansion in EV, *NAA15*‐OE, *NAA15*‐RNAi and *NAA10*‐RNAi plants challenged with *Xoc* strain GDXc267. Scale bar, 1 cm. The red lines indicate lesion length. h) The lesion lengths in EV, *NAA15*‐OE, *NAA15*‐RNAi and *NAA10*‐RNAi plants at 14 days after inoculation with *Xoc* strain GDXc267. i) Phenotypes of the leaves at tillering stage of EV, NAA15‐OE, *NAA15*‐RNAi and *NAA10*‐RNAi plants punch‐inoculated with the *M*. *oryzae* strain 08‐T19. Scale bar, 1 cm. j) The lesion area of (i) was measured at 7 dpi. Statistics: n = 30 per group for c,f); and n ≥ 6 per group for e,h,j). The data shown represent means ± SD. Unpaired, two‐tailed *t*‐tests were performed. ^***^ *p* <0.001.

We have demonstrated that *RESIS* plays a role in BB resistance and interacts with the NatA complex. To investigate the relationships among *RESIS*, NatA, and BB resistance, we generated transgenic plants with *NAA15* (*NAA15*‐KO and *NAA15*‐RNAi) and *NAA10* (*NAA10*‐KO and *NAA10*‐RNAi) knockout and knockdown constructs to conduct phenotypic analyses. The NatA complex is crucial for plant development and stress responses. Consistent with previous studies, we were unable to obtain rice plants with *NAA15* or *NAA10* homozygous knockout mutations.^[^ ^31^, ^32^ ^]^ Thus, we used *NAA15*‐RNAi and *NAA10*‐RNAi transgenic plants for subsequent experiments (Figure S5a,b, Supporting Information). The growth of the *NAA15*‐RNAi and *NAA10*‐RNAi plants was significantly suppressed, affecting plant height and panicle size (**Figure** **4**a,b). Detailed analyses of yield‐related traits in these plants revealed a significant reduction in the number of panicle branches and seeds, as well as yield per plant, compared with those of the control plants, indicating the essential role of NAA15 and NAA10 in maintaining grain yield (Figure 4c; Figure S5c–h, Supporting Information). We also generated *NAA15*‐overexpressing plants (*NAA15*‐OE) (Figure S5i, Supporting Information). No significant developmental differences were detected between the *NAA15*‐OE and control plants (Figure S5j, Supporting Information).

We then evaluated the resistance of the transgenic plants to *Xoo* infection. After treatment with PXO99A, both the *NAA15*‐RNAi and *NAA10*‐RNAi plants presented significant resistance to *Xoo* infection, whereas the *NAA15*‐OE plants presented susceptibility similar to that of the control plants, as determined through quantitative measurements of leaf lesion length and the expression levels of pathogen‐specific genes (Figure 4d,e; Figure S6a, Supporting Information).

The grain yields of the infected plants were also statistically analyzed. After infection, the yield loss of the *NAA15*‐OE plants was higher than that of the *NAA15*‐RNAi and *NAA10*‐RNAi plants (Figure 4c,f; Figure S6b,c, Supporting Information). Since *RESIS* regulates wide‐spectrum resistance, we also assessed the role of NatA in resistance to *M*. *oryzae* and *Xoc*. Similarly, *NatA* knockdown increased the resistance of rice plants to both fungal and bacterial diseases (Figure 4g–j; Figure S6d–j, Supporting Information). These results indicate that the NatA complex functions like *RESIS* during pathogen infection. However, the increased resistance conferred by *NAA15* or *NAA10* knockdown induced developmental defects and yield loss (Figure 4). *RESIS* might regulate pathogen resistance through its effect on the NatA complex, and compared with the NatA complex, *RESIS* presents greater potential for application because its absence enhances resistance without affecting rice yield.

### 2.5 *RESIS* is Essential for the NatA Complex to Function Effectively

**Figure 5:**
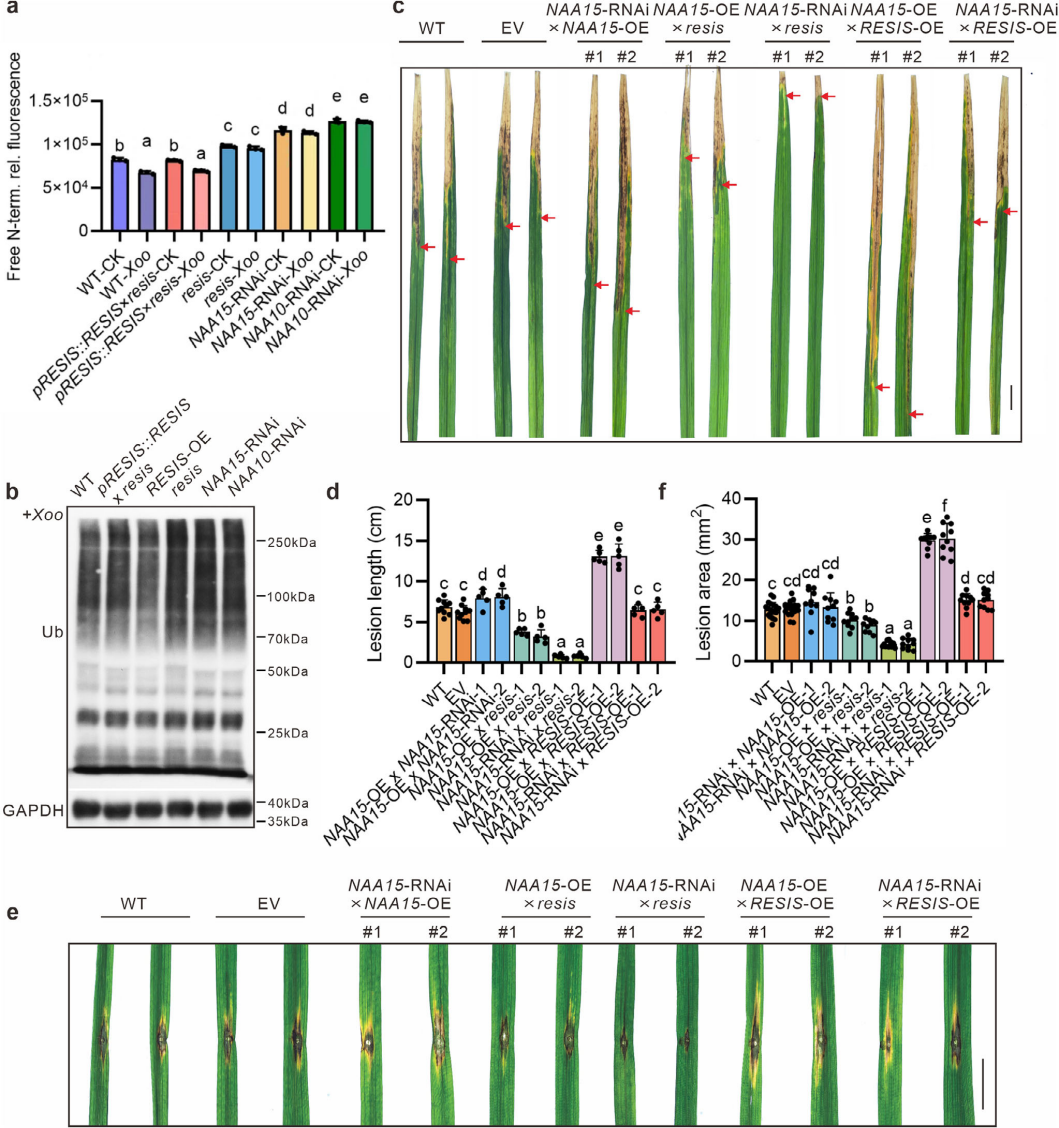
*RESIS* is essential for the function of the NatA complex. a) Quantification of the global number of free N termini with (*Xoo*) or without (CK) *Xoo* inoculation in WT and transgenic plants. Soluble proteins were extracted from the flag leaves. b) Detection of global polyubiquitination levels in *pRESIS*::*RESIS* × *resis*, *resis*, *RESIS*‐OE, *NAA15*‐RNAi and *NAA10*‐RNAi plants. GAPDH served as an internal control. c) Leaves of genetic complementation plants inoculated with *Xoo* strain PXO99A. Photographs were taken 14 days after inoculation. Scale bar, 1 cm. The red arrows indicate lesion length. d) The lesion length in genetic complementation plants at 14 days after inoculation with *Xoo* strain PXO99A. e) Phenotypes of the seedling leaves of genetic complementation plants punch‐inoculated with the *M*. *oryzae* strain 08‐T19. Scale bar, 1 cm. f) The lesion area of (e) was measured at 7 dpi. Statistics: n = 3 per group for a); n ≥ 5 per group for d); and n ≥ 10 per group for f). one‐way ANOVA with Duncan's new multiple range test in a,d, f). Significant differences are indicated by different letters.

*RESIS* has been shown to bind with NAA15 and NAA10 and to exhibit functions similar to those of the NatA complex. Next, we investigated how *RESIS* regulates pathogen resistance through the NatA complex. NatA is the primary enzyme responsible for the N‐terminal acetylation (NTA) of proteins, which serves as a functional signal for protein interaction, folding, and subcellular targeting.^[^ ^31^, ^32^, ^33^ ^]^ To elucidate whether *RESIS* promotes the function of the NatA complex, we assessed the free N‐termini in WT, *NAA15*‐RNAi, *NAA10*‐RNAi, *resis*, and *pRESIS*::*RESIS* × *resis* plants before and after pathogen infection. Briefly, in WT plants, pathogen infection reduced the number of free N‐termini, implying the potential activation of the NatA complex (**Figure** **5**a). A similar pattern was observed in *pRESIS*::*RESIS* × *resis* plants. However, increased free N‐termini were detected in *resis*, *NAA15*‐RNAi, and *NAA10*‐RNAi plants, which is consistent with previous observations of loss‐of‐function mutants of the NatA complex.^[^ ^31^, ^32^ ^]^ Additionally, after pathogen infection, the number of free N‐termini did not decrease in the three transgenic plants, indicating that the function of the NatA complex during the pathogen response was suppressed under *RESIS* knockout conditions, similar to *NAA15* or *NAA10* knockout (Figure 5a).

NTA can protect proteins from degradation by the ubiquitin–proteasome system. The loss of function of the NatA complex is associated with the activation of the ubiquitin–proteasome system.^[^ ^31^, ^33^, ^53^ ^]^ We next examined the polyubiquitination levels of soluble proteins in the transgenic plants. More free N‐termini were associated with increased protein polyubiquitination. During *Xoo* infection, *RESIS*, *NAA15*, or *NAA10* knockdown plants presented significantly increased protein polyubiquitination levels, whereas WT, *pRESIS*::*RESIS* × *resis* and *RESIS‐*OE plants presented lower levels of protein polyubiquitination (Figure 5b). Finally, we examined the regulatory relationship between *RESIS* and NatA by crossing the transgenic plants and analyzing the resistance of their offspring. When *resis* plants were crossed with *NAA15*‐RNAi plants, the offspring presented the highest bacterial and fungal disease resistance, whereas the offspring from the *NAA15*‐OE × *RESIS*‐OE crosses presented the lowest resistance (Figure 5c–f; Figure S6k,l, Supporting Information). The offspring of the *NAA15*‐RNAi × *RESIS*‐OE, *NAA15*‐OE × *resis*, and *NAA15*‐RNAi × *NAA15*‐OE plants presented phenotypes similar to those of WT plants (Figure 5c–f; Figure S6k,l, Supporting Information). Together, these findings suggest that *RESIS* is essential for the ability of the NatA complex to function effectively during the pathogen response.

### 2.6 *RESIS* Enhances the Interaction Strength between NAA15 and NAA10 by Guiding NAA10 to Bind with NAA15

**Figure 6:**
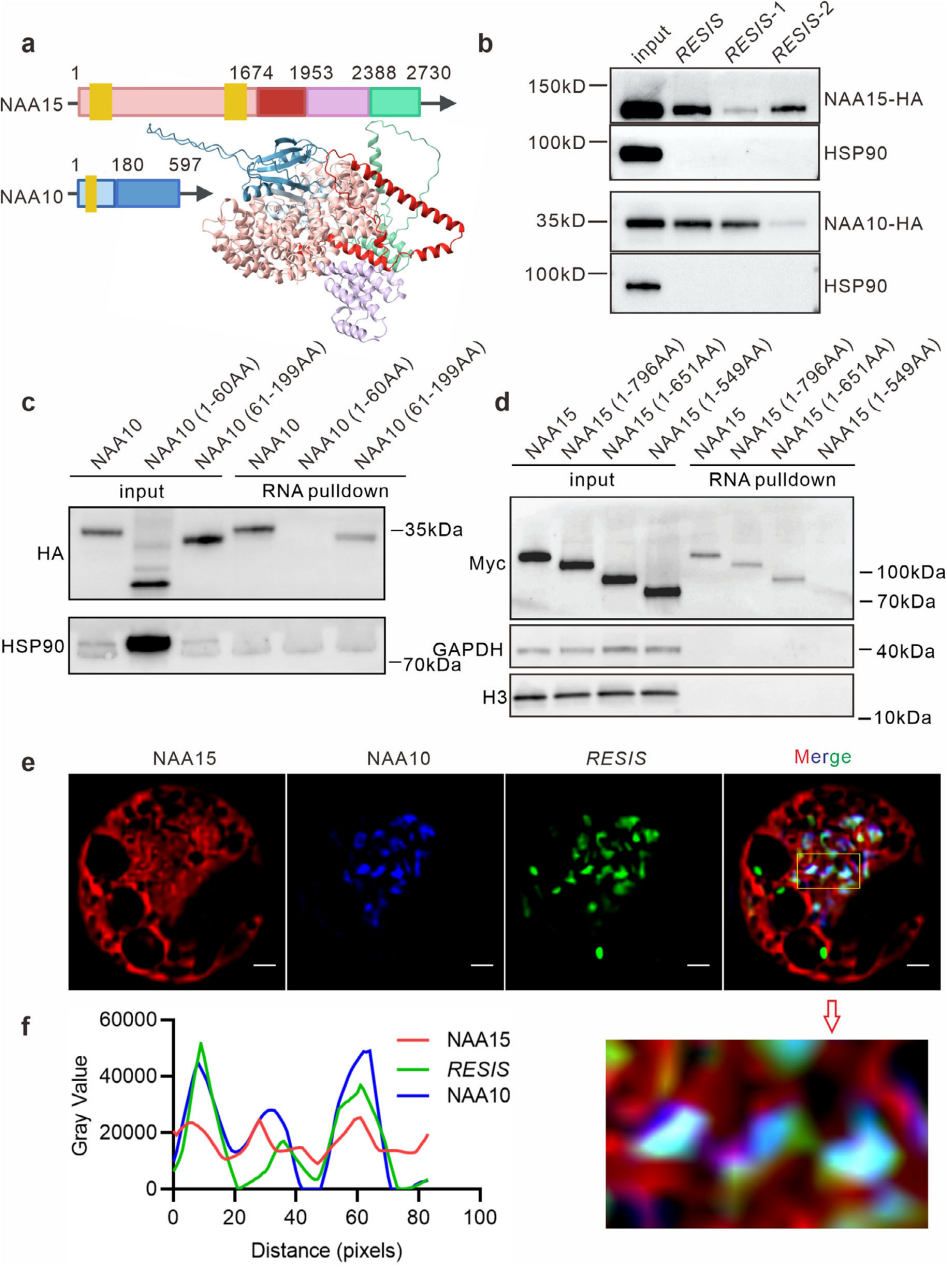
Regulatory mechanism analysis of *RESIS*. a) Schematic diagram of the NAA15 and NAA10 fragments used for binding domain identification. Predicted protein complex structure of NAA15 and NAA10 using AlphaFold3. The distinct colors represent the different fragments of NAA15 and NAA10. The yellow boxes indicate domains that are important for the interaction and activity of the NatA complex. b) RNA pull‐down assay of NAA15 and NAA10 using different *RESIS* fragments. The *RESIS* transcript was divided into two fragments: 1 (1–214 nt) and 2 (215–629 nt). The full‐length sequence and the two fragments were fused to tRSA. HSP90 was used as a control. c) tRSA‐RNA pull‐down assay of full‐length and truncated NAA10 by *RESIS*, as determined by immunoblot analysis with an anti‐HA antibody. HSP90 was used as a control. d) tRSA‐RNA pull‐down assay of full‐length and truncated NAA15 by *RESIS*, as determined by immunoblot analysis with an anti‐myc antibody. GAPDH and H3 were used as controls. e) Colocalization of NAA15, NAA10 and *RESIS* in the cytoplasm, as determined by structured illumination microscopy (SIM). The yellow box indicates the enlarged image in panel f). Scale bars, 1 µm. f) The graph shows the overlap of fluorescence intensity peaks along profiles spanning NAA15, NAA10 and *RESIS*, as indicated in the merged micrograph.

We then analyzed how *RESIS* promoted the function of NatA. lncRNAs reportedly function through diverse mechanisms, including by acting as scaffolds, sponges, and regulators of the transcription and translation of protein‐coding genes or by directly binding to proteins to affect their function.^[^ ^23^, ^24^, ^25^ ^]^ To analyze how *RESIS* affects NAA15 and NAA10 function, we first delineated the binding interface between *RESIS* and NAA15 and between *RESIS* and NAA10 (**Figure** **6**a). We divided *RESIS* into 2 sections according to the RNA structure predictions and performed a tRSA‐RNA pull‐down assay using rice protoplasts co‐transfected with constructs encoding Myc‐tagged NAA15 or HA‐tagged NAA10. Full‐length *RESIS* and *RESIS* fragment 2 (215–629 nt) showed greater binding affinity to NAA15, whereas *RESIS* fragment 1 (1–214 nt) showed greater binding affinity to NAA10 than the other fragments (Figure 6b; Figure S7a, Supporting Information). These results suggest that *RESIS* binds to the NAA15 and NAA10 through distinct regions on its transcript. An in vitro experiment using purified NAA15 and NAA10 proteins further confirmed their interaction with the two regions of *RESIS* (Figure S7b,c, Supporting Information).

NAA10 contains the catalytic domain Gcn5‐related N‐acetyltransferase (GNAT), which is responsible for the acetylation reaction, whereas NAA15 is essential for substrate recognition and ribosome binding through its N‐terminal extension.^[^ ^33^, ^49^ ^]^ Additionally, NAA15 possesses tetratricopeptide repeat (TPR) domains that interact with NAA10, ensuring the proper assembly of the NatA complex.^[^ ^49^ ^]^ On the basis of the interaction regions between NAA15 and NAA10, the ribosome‐binding region of NAA15, and the enzyme activity center of NAA10, we constructed 5 truncated versions of the NAA15 and NAA10 proteins, as shown in Figure 6a. NAA15 fragment 1 (1–549 AA) included the N‐terminus of NAA15 and contained the primary ribosome‐binding region and the NAA10‐binding site. NAA15 fragment 2 (1–651 AA) includes fragment 1 along with an additional helix structure. NAA15 fragment 3 (1–796 AA) extended fragment 2 by incorporating another helix and a disordered region. NAA10 fragment 1 (1–60 AA) included the N‐terminal of NAA10 and contained the NAA15‐binding core‐site. NAA10 fragment 2 (61–199 AA) included the C‐terminal of NAA10 (Figure 6a; Figure S7d, Supporting Information). These truncated proteins were used to identify the specific binding regions of NAA15 and NAA10 to *RESIS*. Our results indicated that *RESIS* bound to the helix region of NAA15 fragment 2 and to NAA10 fragment 2 (Figure 6c,d). An in vitro experiment using purified fragments of NAA15 and NAA10 confirmed their specific interaction regions with *RESIS* (Figure S7e,f, Supporting Information).

The structures of NAA15 and NAA10, together with their interactions, were predicted through Alphafold3^[^ ^54^ ^]^ and ChimeraX.^[^ ^55^ ^]^ Interestingly, the *RESIS* binding sites of NAA15 and NAA10 are both present on the same side of the surface of the NatA complex, implying that *RESIS* might interact with the exposed region of NAA15 and NAA10 to enhance their interaction and function (Figure 6a). To test this hypothesis, we performed enhanced single RNA imaging to track the subcellular localization of *RESIS* together with the NatA complex. In brief, 12 × MS2 was placed onto the 5′ terminal of *RESIS*, and up to 576 sfGFP were tethered to a single *RESIS* transcript through the MSCP‐Suntag‐scFv‐sfGFP interaction. NAA15‐mCherry and NAA10‐BFP were co‐expressed in rice protoplast cells. Structured illumination microscopy (SIM) revealed partial overlap among *RESIS*, NAA10, and NAA15 (Figure 6e). Notably, the fluorescence signal of NAA15 formed the outermost layer, encapsulating both the NAA10 and *RESIS* signals, with partial overlap observed between NAA15 and *RESIS* (Figure 6e). Additionally, NAA10 showed a stronger overlap with *RESIS*, and its signal was exclusively detected in the presence of *RESIS* (Figure 6e). This spatial distribution of the fluorescence signals for *RESIS*, NAA15, and NAA10 aligned well with the molecular interaction model established through our experimental verification (Figure 6f). Importantly, this also reveals a novel organizational model of the NatA complex, in which *RESIS* guides NAA10 to interact with NAA15.

**Figure 7:**
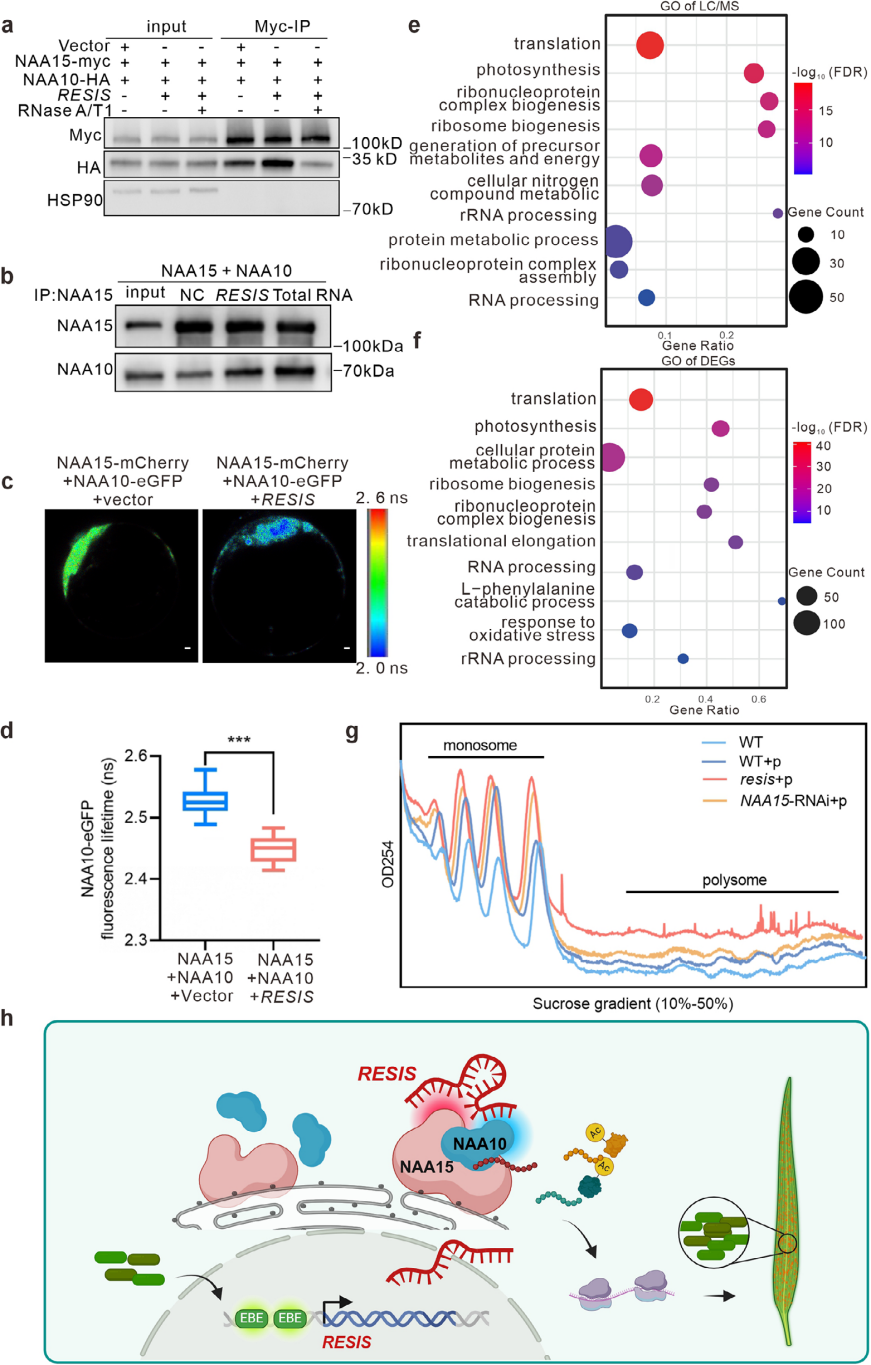
Pathways regulated by the *RESIS*‐NatA complex. a) Co‐immunoprecipitation (co‐IP) analysis of the interaction strength between NAA15 and NAA10, with or without *RESIS* overexpression or RNase A/T1 treatment in protoplasts. b) Purified NAA15 co‐IP with NAA10 with or without *RESIS* or total RNA in vitro. c,d) Representative FLIM images (c) and data (d) showing the fluorescence lifetime of eGFP recorded 16 h after *pRTV*‐*NAA10*‐eGFP and *pRTV*‐*NAA15*‐mCherry were co‐expressed with *pRTV* or *pRTV*‐*RESIS* in protoplasts. The scale varies from the shortest lifetime of 2.0 ns to the longest lifetime of 2.6 ns. Scale bars, 1 µm. e) GO enrichment analysis of proteins with reduced N‐terminal acetylation in both *resis* and *NAA15*‐RNAi plants. f) GO enrichment analysis was performed on DEGs that are common targets of *RESIS* and NAA15. g) Polysome profiling experiments detected changes in translation efficiency before and after pathogen induction (+p) in WT, *resis*, and *NAA15*‐RNAi plants. h) Working model of the *RESIS* module: Upon pathogen invasion, *RESIS* is activated through the EBEs on its promoter region. The *RESIS* transcript then interacts with both NAA15 and NAA10 via two distinct regions, guiding NAA10 to bind with NAA15 and thereby activating the NatA complex. This activation leads to the N‐terminal acetylation of proteins involved in translation, followed by hijacking the translation machinery to favor pathogen growth. Notably, *RESIS* knockout enhances the resistance of rice plants to pathogen invasion without growth inhibition. Statistics: n = 20 per group for d). Data shown represent the mean ± SD. Unpaired, two‐tailed *t*‐tests were performed. ^***^ *p* <0.001.

As *RESIS* was shown to interact with NAA15 and NAA10 at the surface of the assembled NatA complex and is required for the normal function of the NatA complex, we speculated that *RESIS* is essential for NatA complex assembly. With respect to the binding affinity between NAA15 and NAA10, their interaction strength significantly increased in the presence of *RESIS* in a concentration‐dependent manner both in vitro and in vivo (**Figure** **7**a,b). Furthermore, a fluorescence lifetime imaging with Förster resonance energy transfer (FLIM–FRET) assay was performed to evaluate the interaction strength between NAA15 and NAA10 in the presence or absence of *RESIS*. The donor NAA10‐eGFP and receptor NAA15‐mCherry were co‐expressed in rice protoplasts, and the fluorescence lifetime was measured by FLIM–FRET; upon co‐expression with *RESIS*, the mean fluorescence lifetime of the donor decreased from *t* = 2.525 ns to *t* = 2.448 ns, suggesting that *RESIS* promoted the interaction between NatA complex members in vivo (Figure 7c,d).

Collectively, our results suggest that *RESIS* guides the interaction between NAA15 and NAA10, enhancing the activity of the NatA complex and positively regulating its role in pathogen resistance.

### 2.7 *RESIS* Underwent Exaptation during Domestication to Promote N‐Terminal Acetylation and Regulates Translation during Pathogen Infection with the NatA Complex

We identified *RESIS* as a lncRNA that was conserved during rice domestication. We next investigated whether the regulatory effect of *RESIS* on the NatA complex during pathogen invasion is an evolutionarily conserved pathway. The NatA complex is highly conserved across varieties, and the *RESIS* binding site is also conserved in *Arabidopsis* ^[^ ^31^, ^32^, ^33^ ^]^ (Figure S8a,b, Supporting Information). Interestingly, the analyses of the conservation and expression variation in *RESIS* from wild rice to cultivated rice revealed that the 3′ end of the lncRNA evolved. In *O*. *rufipogon*, *RESIS* is 299 nt in length and, compared with *RESIS* in *O*. *japonica*, lacks 329 nt at the 3′ end, which importantly serves as the binding site for NAA15 and is essential for the regulatory function of *RESIS* on the NatA complex. In addition, the EBEs in the promoter region of *RESIS* were disrupted in wild rice (Figure S8c, Supporting Information). But *RESIS* showed high sequence conservation across different cultivars, with both cultivars harboring EBEs (Figure S8c, Supporting Information). Thus, the regulatory effect of lncRNA on the NatA complex is likely a consequence of rice domestication.

We next investigated how the *RESIS*‐NatA module plays a role in pathogen resistance. Although previous studies have demonstrated the importance of the NatA complex in various abiotic stress responses,^[^ ^31^, ^32^, ^56^ ^]^ its role in pathogen resistance remains unexplored. Therefore, we sought to identify the target proteins of the *RESIS*‐NatA module. As previously described,^[^ ^57^ ^]^ we used a high‐confidence large‐scale N‐terminal protein acetylation method. We analyzed and identified the N‐acetylated peptides that were significantly reduced in *resis* and *NAA15*‐RNAi plants compared to WT plants using a label‐free quantification method (Table S4, Supporting Information). The N‐terminal acetylation of 226 proteins was significantly down‐regulated in both *resis* and *NAA15*‐RNAi plants, which are considered as the targets of the *RESIS*‐NatA module (Table S4, Supporting Information). These proteins were significantly enriched in translation‐related pathways, suggesting a potential regulatory role of the *RESIS*‐NatA complex in translation (Figure 7e; Table S5, Supporting Information). Several proteins, including OsEF1α1,^[^ ^58^ ^]^ OsGF14C,^[^ ^59^ ^]^ OsNDPK4,^[^ ^60^ ^]^ and LC7,^[^ ^61^ ^]^ which have been reported to promote pathogen invasion, are targeted by this module. Additionally, seven transcription factors (TFs), such as MYB, bZIP, and C2H2, which are wildly involved in disease‐related processes,^[^ ^62^, ^63^, ^64^, ^65^ ^]^ were also identified as targets (Table S4, Supporting Information). To investigate the downstream target genes regulated by this module, we performed transcriptome analysis of flag leaf samples from WT, *resis*, and *NAA15*‐RNAi plants, with three biological replicates for each. Compared to WT plants, 528 differentially expressed genes (DEGs) exhibited similar expression patterns in both *resis* and NAA15‐RNAi plants and were identified as their common targets (Figure S8d, Table S6, Supporting Information). These DEGs were significantly enriched in ribosome assembly and translation‐related pathways, and included 55 diseased‐related genes (Figure 7f; Tables S6 and S7, Supporting Information). We predicted the upstream regulatory elements of these DEGs, and found that 38% of them contained regulatory elements of the TFs that were identified as the N‐ terminal acetylation targets of the *RESIS*‐NatA module. Of the 55 disease‐related DEGs, 36 were potential targets of these TFs (Figure S8d, Table S6, Supporting Information). These results suggest that the NatA‐*RESIS* complex likely influences the transcription of downstream gene by regulating protein acetylation.

To explore the role of the *RESIS*‐NatA complex in ribosome assembly and translation, we conducted polysome profiling using WT, *resis*, and *NAA15*‐RNAi plants. Pathogens are known to affect host protein synthesis, and the host mounts a defense response by controlling the translation process, leading to an increase in the transcription of immune genes.^[^ ^66^ ^]^ Our results revealed that translation increased after pathogen infection in WT plants and that translation was further increased by *RESIS* or *NAA15* knockdown, suggesting that the *RESIS*‐NatA complex regulates resistance through the modulation of translation (Figure 7g). Together, our findings demonstrate that the *S* lncRNA *RESIS* promotes the assembly of the NatA complex, thereby regulating N‐terminal acetylation and translation in the response of plants to pathogens (Figure 7h). Importantly, *RESIS* can be targeted to enhance plant resistance without compromising growth, highlighting the potential of *S* lncRNAs as targets for genome editing in future crop improvement.

## 3 Discussion

Improving crop resistance without yield loss is a challenge often encountered in crop breeding. Compared with that of wild rice, the yield of cultivated rice is significantly greater; however, the survival advantage of cultivated rice decreases significantly during cultivation, as cultivated rice is susceptible to various diseases.^[^ ^8^, ^9^, ^67^ ^]^ The genomic editing of resistance‐related genes can improve plant survival under pathogen infection, but it often comes at the cost of suppressing plant growth.^[^ ^3^, ^13^, ^68^, ^69^, ^70^ ^]^ Therefore, there is an urgent need to develop efficient strategies to enhance the resistance of cultivated rice to diseases without introducing undesirable traits. In this study, we reported a novel strategy to enhance the broad‐spectrum resistance of rice plants to pathogens without compromising growth and grain yield by knocking out an *S* lncRNA, which is expressed at a low‐level during development and almost does not affect the normal growth of rice plants. This lncRNA, named *RESIS*, is induced by pathogens and is considered an *S* lncRNA. Upon pathogen invasion, *RESIS* is induced through its EBE motif and subsequently guides the interaction between the two core components of the NatA complex, NAA15 and NAA10. While NAA15 facilitates pathogen invasion, it is also crucial for the normal growth of rice plants. Therefore, enhancing resistance by knocking down *RESIS*, rather than directly targeting the NatA complex, offers a more advantageous approach for crop breeding.

lncRNAs are known for their highly tissue‐specific expression and rapid evolution, which led to their initial classification upon discovery.^[^ ^23^, ^24^ ^]^ Furthermore, compared with protein‐coding genes, most lncRNAs are more sensitive to environmental stimuli.^[^ ^25^, ^26^, ^27^ ^]^ These characteristics lead to the intriguing hypothesis that a class of lncRNAs plays crucial roles in sensing environmental changes without regulating major developmental processes. Consistently, recent studies have demonstrated that lncRNAs can fine‐tune regulatory roles that are essential for organisms.^[^ ^26^, ^71^ ^]^ In this study, we identified *RESIS* as an lncRNA that is expressed at low levels during development and is activated by pathogens. Knocking out *RESIS* significantly enhanced resistance to pathogens without causing developmental defects. *S* lncRNAs, which are pivotal in sensing environmental stress, represent ideal targets for genomic editing in crop breeding, addressing the critical challenge of enhancing resistance without sacrificing yield. *RESIS* exerts its function by regulating ribosome activity and translation during pathogen invasion. A similar mechanism involving lncRNAs has been reported in plants. For instance, the rice lncRNA *cis‐NATPHO1.2* is upregulated under phosphorus (Pi) deficiency, enhances its cognate PHO1.2 mRNA translation by promoting 80S ribosome formation, and contributes to Pi homeostasis.^[^ ^72^ ^]^ Interestingly, global analysis of ribosome‐associated RNAs revealed a considerable number of lncRNAs with ribosome footprints.^[^ ^73^ ^]^ Some of these lncRNAs may have translation potential, generating micropeptides, such as lncRNA *ENOD40* (*Early Nodulin 40*)^[^ ^74^, ^75^ ^]^ or miPEPs encoded by miRNA precursors.^[^ ^76^ ^]^ It remains to be explored whether others act as translation regulators, making them an interesting area for further study.

The N‐terminal acetylation of proteins catalyzed by the NatA complex is a conserved mechanism for regulating protein function and metabolism in both animals and plants.^[^ ^77^ ^]^ Loss of function of the NatA complex can lead to severe developmental defects.^[^ ^31^, ^32^ ^]^ NAA15 and NAA10 are the major components of the NatA complex; NAA15 has been shown to interact with ribosomes, while NAA10 binds to NAA15 and exhibits catalytic activity.^[^ ^31^, ^32^ ^]^ HYPK has been identified as a regulatory component, but whether other regulatory factors, particularly RNA components, are involved remains unclear.^[^ ^31^, ^32^ ^]^ We found that *RESIS* interacts with both NAA15 and NAA10 through different regions, and super‐resolution microscopy revealed that *RESIS* may act as an anchor, facilitating the binding of NAA10 to NAA15. This interaction is crucial for the function of the NatA complex during pathogen infection. Therefore, RNA components could play important roles in this complex. Interestingly, the NatA complex exhibits dual effects in responding to different environmental cues. For instance, it is activated by heat stress and plays a positive role in heat resistance.^[^ ^78^ ^]^ The expression of *RESIS* during normal development is extremely low, but it is also activated by heat stress in addition to pathogen infection. The induction of *RESIS* by heat stress may enhance the activity of the NatA complex, thereby increasing plant resistance to heat stress. Therefore, *RESIS* may have been retained during the domestication of rice.

LncRNAs are known for their diverse regulatory mechanisms. One conserved mechanism is their function as scaffolds or decoys by interacting with multiple proteins. For example, in *A. thaliana, APOLO* (*auxin‐regulated promoter loop*) interacts with several proteins, including LHP1, VIM1, and WRKY42, to coordinate histone and DNA methylation over target genes.^[^ ^79^, ^80^, ^81^, ^82^ ^]^ *ASCO* (*Alternative Splicing Competitor*) interacts with NSRs, as well as other spliceosome core components such as PRP8a and SmD1b, influencing protein distribution in cellular compartments and alternative splicing.^[^ ^83^, ^84^ ^]^ *RESIS* has been shown to act as a scaffold, promoting the interaction between NatA complex members in the cytoplasm. Additionally, the binding region of *RESIS* on NAA15 was acquired in cultivated rice during dominance. This alteration allows the lncRNA to modulate the activation of the NatA complex only in cultivated rice, whereas this regulatory function is absent in wild rice. The amplification of sequences in lncRNAs during evolution is likely a common event that facilitates the emergence of new protein‐binding sites.^[^ ^85^, ^86^ ^]^ This, in turn, may lead to novel lncRNA–protein regulatory relationships with physiological functions. Therefore, we propose that these newly evolved lncRNA–protein interaction modules, particularly lncRNAs, could serve as effective targets to balance development and resistance in crop breeding. Overall, our study presents a promising biotechnology strategy to enhance broad‐spectrum resistance in crops without compromising yield or causing developmental deficiencies while also highlighting future applications of molecular design breeding utilizing *S* lncRNAs as genomic editing targets.

## 4 Conclusion

In summary, our results elucidate the mechanistic framework by which the susceptibility lncRNA *RESIS* orchestrates the assembly of the NatA complex to regulate N‐terminal acetylation and global translation during pathogen invasion. Knocking out *RESIS* confers broad‐spectrum resistance to both fungal and bacterial pathogens without compromising plant growth or yield, thereby effectively circumventing the classical growth–defense trade‐off. These findings offer novel insight into the functional role of susceptibility lncRNAs in plant immunity and present a promising, practical molecular breeding strategy for developing high‐yield, disease‐resistant crop varieties.

## 5 Experimental Section

### Plant Growth Conditions, Generation of Transgenic Rice Plants, and Phenotype Analysis

The *japonica* rice (*Oryza sativa*) cultivar ‘Zhonghua 11′ (ZH11) was used in this study. The growth conditions and generation of transgenic plants were conducted according to Zhang et al.^[^ ^87^ ^]^ Briefly rice seeds from the control plants and the transgenic plants imbibed in darkness for 2 d at 30 °C and then were grown for ≈20 d in a soil seed bed at 28 °C, 70% humidity (12 h light/12 h dark), and then the seedlings were transplanted to a field in Guangzhou, China (23°08′N, 113°18′E), where the growing season extends from late April to late September. The mean minimum temperature range was 22.9–25.5 °C, and the mean maximum temperature range was 29.7–32.9 °C. The day length ranged from 12–13.5 h. Plants were cultivated using routine management practices. The *RESIS* T‐DNA insertion mutant is obtained from the Rice Mutant Database of Pohang University of Science and Technology.^[^ ^88^ ^]^ The *RESIS* knockout mutants were generated using CRISPR/Cas9‐based genome editing technology.^[^ ^89^ ^]^ The *RESIS* and *NAA15* overexpression transgenic plants were constructed using the *pRHV*‐vector.^[^ ^90^ ^]^ The *pRNAi*‐35S binary vector, which has been described by Zhang et al. was used to generate the *NAA15*‐RNAi and *NAA10*‐RNAi mutant.^[^ ^87^ ^]^ For complementation, genomic DNA containing the *RESIS* promoter as well its coding region was amplified and inserted into the *pRHV* vector. The resulting construct *pRHV*‐*pRESIS*::*RESIS* was transformed into *resis* calli via *Agrobacterium tumefaciens*‐mediated transformation. The T3 generations of the transgenic plants were used for phenotypic analyses. The primers are listed in Table S8 (Supporting Information).

### RNA Extraction, and RT‐qPCR

For quantification of gene expression, total RNAs were extracted from different tissues using the TRIzol reagent (Invitrogen). 1 µg RNA was reverse‐transcribed into cDNA using oligo (dT) primer and SuperScript III reverse transcriptase (Invitrogen). RT‐qPCR was carried out using SYBR Premix Ex Taq (Takara). The RT‐qPCR was performed according to the manufacturer's instructions (Takara), and the resulting melting curves were visually inspected to ensure the specificity of the product detection. Quantification of gene expression was performed using the comparative *C*t method. Experiments were performed in triplicate, and the results are represented as the mean ± standard deviation (SD). The primers are listed in Table S8 (Supporting Information).

### Protein Expression and Purification

Expression plasmids for His‐Avrxa27CRR, His‐SUMO‐*NAA15*, His‐SUMO‐NAA15 (1–549 AA), His‐SUMO‐NAA15 (550–909 AA), His‐MBP‐NAA10, His‐MBP‐NAA10 (1–60 AA), and His‐MBP‐NAA10 (61–199 AA) in pET systems were individually transformed into E. coli expression strain BL21 [Transetta (DE3) chemically competent cells (Vazyme)]. A single colony was inoculated in 5 mL LB media supplemented with 100 mg L^−1^ kanamycin at 180 rpm, 37 °C for 14–16 h. The culture was diluted 100‐fold into 100 mL LB media supplemented with 100 mg L^−1^ kanamycin at 180 rpm, until the optical density (OD600) of bacterial suspension reached 0.4–0.6. IPTG (0.25 mm) was added to the bacterial suspension. After overnight incubation at 150 rpm at 20 °C for 14 h, cell pellets were harvested by centrifugation (10 000 rpm, 10 min, 4 °C), washed twice, and resuspended in 25 mL binding buffer (20 mm Tris‐HCl pH 7.4, 500 mm NaCl, 20 mm imidazole, 1 mm PMSF). Cell pellets were further sonicated for 40 min (300 W, 3 s on / 9 s off) on ice. After centrifugation at 12 000 rpm for 15 min at 4 °C, the supernatant cell lysates were filtered through a 0.22 µm filter and then certain proteins were purified with ÄKTA pure chromatography system (Cytiva) using HisTrap HP (Cytiva) at 4 °C. Then the protein further purified over the gel filtration chromatography (Superdex 200 Increase; Cytiva) equilibrated with storage buffer (20 mm Tris‐HCl pH 7.5, 100 mm NaCl, and 10% Glycerol) and the protein was store at −80 °C. The concentration of purified protein was determined by using the Bradford Protein Assay Kit (Takara) and checked by Coomassie Brilliant Blue staining.

### EMSA

Protein purification was performed as previously described. The oligonucleotides and RNA probe were synthesized and labeled with Cyanine5 (Cy5). Double‐stranded oligonucleotides were generated by mixing equal amounts of the complementary single‐stranded oligonucleotides and heating for 2 min at 95 °C, then cooling down to 25 °C. Cy5‐labeled probes (20 ng) were incubated with protein in binding buffer (10 mm Tris‐HCl pH 7.5, 50 mm KCl, 1 mm EDTA, 5 mm MgCl_2_, 1 mm DTT, 50 ng µL^−1^ poly (dI‐dC), 2.5% glycerol, and 0.05% NP‐40) for 20 min at room temperature. For the competition reaction, different concentrations of non‐labeled probes were mixed with the labeled probes. The DNA reaction mixture was loaded onto a 6% native polyacrylamide gel, while the RNA reaction mixture was loaded onto a 4% native polyacrylamide gel, and both were run at 4 °C. The DNA and RNA shift were detected by developing the Cy5 signal using a laser scanning imaging system (ChemiDoc MP, Bio‐Rad) according to the manufacturer's instructions. Oligonucleotides used for the EMSA are given in Table S8 (Supporting Information).

### Pathogen Inoculation Experiments

The *Xoo* strain PXO99A and *Xoc* strain GDXc267 were cultured on a peptone sucrose agar (PSA) medium at 28 °C for 3 days. The bacteria were collected and then suspended in sterilized water at a concentration of OD600 = 1.0. PXO99A was inoculated by the leaf‐clipping method, whereas GDXc267 was performed the infiltration on the underside of the leaves by pressing the mouth of the syringe on the leaf surface. DNA was extracted from leaf segments at 14 dpi to quantify bacterial *hrpC* and rice *EF1α* gene copy numbers via qPCR. Lesion length was measured at 14 dpi. Blast fungal inoculation was conducted as previously described.^[^ ^13^, ^69^ ^]^ Briefly, for rice blast inoculation, *M*. *oryzae* (08‐T19) spore concentration was adjusted to ≈1 × 10^5^ per mL. Two‐week‐old rice seedlings were spray‐inoculated with blast spore suspensions in a dew growth chamber. Leaves of seedlings and tillering plants were punch‐inoculated. At 7 dpi, relative fungal growth was calculated by DNA‐based quantitative PCR (qPCR) using the threshold cycle value (*C*_T_) of *M*. *oryzae Pot2* transposon DNA against the *C*_T_ of rice genomic *ubiquitin* gene (*LOC_Os03 g13170*). PCR primers are listed in Table S8 (Supporting Information). All fungal and bacterial infections were repeated independently at least three times. Samples from two or three transgenic lines were pooled for qPCR analysis and lesion phenotyping (length and area).

### Nuclear, Cytoplasmic, and Endoplasmic Reticulum Fractionation

Cytoplasmic and nuclear fractionation was performed as described by Wang et al.^[^ ^91^ ^]^ with modifications. Plant leaves were ground in liquid nitrogen and homogenized in lysis buffer. The homogenate was filtered, and the flow‐through was centrifuged at 1500 g for 10 min at 4 °C. The supernatant was further centrifuged at 10000 g for 10 min at 4 °C to obtain the cytosolic fraction. The pellet was washed with NRBT buffer, resuspended in NRB2, overlaid on NRB3, and centrifuged at 10000 g for 15 min at 4 °C to isolate the nuclear fraction. Isolation and enrichment of ER were carried out using Minute Plant ER Enrichment Kit (invent) according to the manufacturer's protocol.

### RNA In Situ Hybridization and Immunofluorescence

Cy3‐labeled oligonucleotide probes specifically targeting *RESIS* were designed and synthesized by RiboBio corporation. Root hair cells of WT and *RESIS*‐OX were collected fixed with 4% paraformaldehyde for 30 min and then permeabilized with 0.5% Triton X‐100 for 30 min. Cells were incubated with Cy3‐labeled FISH probes dissolved by 50% formamide in 2 × SCC at 37 °C overnight. After hybridization, the nuclei were counterstained with DAPI. Cells were observed on a Zeiss7 DUO NLO LSM880 confocal laser microscope (Carl Zeiss).

### 5′ RACE and 3′RACE

Total RNA from the WT spikelet was extracted using liquid nitrogen and TRIzol reagent (Invitrogen) according to the manufacturer's guidelines. The 5′‐ and 3′‐ends of cDNA were acquired using a 5′‐FULL RACE Kit with TAP (Takara) and 3′‐FULL RACE Core Set with PrimeScript RTase (Takara), respectively, according to the manufacturer's instructions. PCR products were obtained and then cloned into pEASY‐Blunt (TransGen Biotech) for further sequencing. The primers of RACE are listed in Table S8 (Supporting Information).

### Rice Protoplast Transient Transformation

The protoplasts were isolated based on the method reported by Zhang et al. with slight modifications.^[^ ^92^ ^]^ Briefly, two‐week‐old rice shoots were used for protoplast isolation. ≈100 rice plants were cut into ≈0.5 mm strips with a propulsive force using sharp razors. The strips were incubated in enzyme solution (1.5% cellulose RS, 0.75% macerozyme R‐10, 0.6 m mannitol, 10 mm MES pH 5.7, 10 mm CaCl_2_, and 0.1% BSA) for 4–5 h in the dark with gentle shaking. Following enzymatic digestion, an equal volume of W5 solution (154 mm NaCl, 125 mm CaCl_2_, 5 mm KCl, and 2 mm MES pH 5.7) was added, and samples were shaken for 30 min. Protoplasts were released by filtering through a 40 µm nylon mesh into round‐bottom tubes and were washed three to five times with W5 solution. The pellets were collected by centrifugation at 150 g for 5 min in a swinging bucket. After washing with W5 solution, the pellets were then resuspended in MMG solution (0.4 m mannitol, 15 mm MgCl_2,_ and 4 mm MES pH 5.7) at a concentration of 2 × 10^6^ cells mL^−1^. Aliquots of protoplasts (200 µL) were transferred into a 2 mL round‐bottom microcentrifuge tube and mixed gently with 20 µg plasmid DNA. Transfected protoplasts were collected by centrifugation for 5 min at 100 × g, resuspended, and then incubated at 28 °C in the dark for 20 h.

### Subcellular Localization

Both the Suntag^[^ ^93^ ^]^ system and the MS2 system were modified. Briefly, 2 × MSCP‐24 × GCN4, scFv‐sfGFP, and 12 × MS2‐*RESIS* was fused to *pRTV* ^[^ ^90^ ^]^ with no tag, NAA15 was fused to *pRTV*‐mCherry, and *pRTV*‐eGFP, NAA10 was fused to *pRTV*‐BFP. *pRTV*‐2 × MSCP‐24 × GCN4, *pRTV*‐scFv‐sfGFP, *pRTV*‐12 × MS2‐*RESIS*, *pRTV*‐NAA15‐mCherry and *pRTV*‐NAA10‐BFP were co‐expressed into protoplasts. Images were acquired on a Multi‐SIM (Multimodality Structured Illumination Microscopy) imaging system (NanoInsights‐Tech Co., Ltd.) equipped with an Objective Plan‐Apochromat 63x/1.40 Oil M27 (ZEISS). *pRTV‐*NAA15‐eGFP was co‐expressed with the nuclear mCherry marker HY5^[^ ^94^ ^]^ and the endoplasmic reticulum (ER) mCherry marker.^[^ ^95^ ^]^ Confocal laser scanning microscopy was performed using Zeiss7 DUO NLO LSM880 (Carl Zeiss).

### TriFC Assays

The BiFC vectors *pRTV*‐VN and *pRTV*‐VC were modified to generate TriFC assays vectors with an MS2 system.^[^ ^96^ ^]^ Briefly, 2 × MSCP was fused to *pRTV*‐VN, NAA15 was fused to *pRTV*‐VC, 12 × MS2‐*RESIS* or 12 × MS2 was fused to *pRTV* with no tag. PEG‐mediated protoplast transfections were performed as previously described. Protoplasts were observed using HIS‐SIM (High Intelligent and Sensitive SIM) provided by Guangzhou CSR Biotech Co. Ltd. Images were acquired using a 100×/1.5 NA oil immersion objective (Olympus).

### RNA Pull‐Down Assay

tRSA‐RNA pull‐down assays were performed based on previous publications with modifications.^[^ ^48^ ^]^ *RESIS* full‐length sense, *RESIS*‐1 (1–214 nt), and *RESIS*‐2 (215–629 nt) was cloned into the pEASY‐Blunt plasmid and its internal sequences were cloned into the pEASY‐Blunt plasmid containing the 5′ terminal tRSA tag. The plasmids were used as templates to in vitro transcribe RNA products using the TranscriptAid T7 High Yield Transcription Kit (Thermo Fisher Scientific). Then the RNA products were purified using the GeneJET RNA Purification Kit (Thermo Fisher Scientific). 50 pmol RNA per reaction system were denatured 5 min at 85 °C and slowly cooled down to room temperature. The powdered plant tissues, protoplast cells, or purified proteins were collected and resuspended in an appropriate volume of extraction buffer containing RNase inhibitor (Invitrogen) and a cocktail of protease and phosphatase inhibitors (Thermo Fisher Scientific). The RNA pulldown assay was performed using the Pierce Magnetic RNA‐Protein Pull‐Down Kit in accordance with the manufacturer's instructions (Thermo Fisher Scientific). The interacted proteins were determined by Mass spectrometry or Western blotting. The list of antibodies is provided in Table S9 (Supporting Information).

### Immunoprecipitation

In the Co‐IP experiment with NAA15 and NAA10 in vivo and in vitro, anti‐Myc or anti‐NAA15 was used, and the samples were boiled after vigorous washing for three times. The proteins from protoplasts for Co‐IP were lysed with Co‐IP lysis buffer supplemented with a cocktail of protease and phosphatase inhibitors (Thermo Fisher Scientific). Finally, all samples were suspended in loading buffer and then denatured for 5 min at 100 °C, separated via SDS‐PAGE, transferred to PVDF membranes, and blotted. For RNA immunoprecipitation assays, protoplasts co‐expressing *pRHV*‐NAA15‐Myc, *pRHV*‐NAA15‐Myc (1–549 AA), *pRHV*‐NAA15‐Myc (550–909 AA) with *pRHV*‐*RESIS*, or *pRHV*‐NAA10‐HA, *pRHV*‐NAA10‐HA (1–60 AA), *pRHV*‐NAA10‐HA (61–199 AA) with *pRHV*‐*RESIS*, were lysed in RNA immunoprecipitation extraction buffer containing RNase inhibitor (Invitrogen) and a cocktail of protease and phosphatase inhibitors (Thermo Fisher Scientific). Myc, HA, or immunoglobulin G (IgG) antibodies (2 µg) were incubated with lysates for 1 h at 4 °C, followed by overnight incubation with Protein A/G magnetic beads (Thermo Fisher Scientific). RNA extraction from the beads was further collected by using TRizol after proteinase K (New England Biolabs) treatment. Reverse transcription and qPCR were performed as previously described.

### TurboID‐Mediated Proximity Labeling of lncRNA

The *pRHV*‐TurboID vectors were modified to generate lncRNA TurboID assays vectors with an MS2 system. Briefly, 2 × MSCP was fused to *pRHV*‐TurboID, 12 × MS2‐*RESIS* was fused to *pRHV*‐TurboID without the sequence of TurboID. Aliquots of protoplasts (800 µL) were transferred into a 5 mL round‐bottom microcentrifuge tube and mixed gently with 80 µg plasmid DNA. Transfected protoplasts were collected by centrifugation for 5 min at 100 g, resuspended, and then incubated at 28 °C in the dark for 20 h. 50 µm biotin was added to incubate for an additional 30 min. The samples were then harvested to extract total proteins with ice‐cold lysis buffer (50 mm Tris‐HCl pH 7.5, 150 mm NaCl, 0.5% (w/v) sodium deoxycholate, 0.1% (w/v) SDS, 1 mm EDTA, 1 mm DTT, 1 mm PMSF, 10 µg mL^−1^ leupeptin, and 1 × protease inhibitor cocktail). The purification and enrichment of biotin‐labeled proteins were performed according to the protocols described previously.^[^ ^97^ ^]^ Finally, the purified proteins were digested by trypsin on streptavidin beads and subjected to liquid chromatography with tandem mass spectrometry analysis.

### Silver Staining and Mass Spectrometry

Equal amounts of the retrieved proteins obtained from the RNA pull‐down assay were loaded on a 10% SDS–PAGE gel. The gel was further stained using the Pierce Silver Stain for Mass Spectrometry kit (Thermo Fisher Scientific) in accordance with the manufacturer's instructions. The specific bands were cut for mass spectrometry analysis.

### Mass Spectrometry Analyses

Soluble leaf proteins from 2‐week‐old WT, *resis*, and *NAA15*‐RNAi seedlings were extracted using the extraction buffer (50 mm Tris‐HCl pH 7.5, 150 mm NaCl, 15 mm MgCl_2_, 1 mm EDTA, 10% glycerol, 1% Triton X‐100, 2 mm PMSF, and 1× complete protease inhibitor cocktail) for quantification of N‐terminal protein acetylation. As previously described,^[^ ^57^ ^]^ 1 mg of extracted proteins was precipitated by adding four times the sample volume of cold acetone and dried. The precipitate was subsequently dissolved in 6 m Guanidine‐HCl, 50 mm Tris‐HCl (pH = 8), and 4 mm DTT, denatured at 95 °C for 15 min. After the sample cooled to room temperature, iodoacetamide (55 mm) was added for alkylation at room temperature for 1 h. Proteins were precipitated by adding four times the sample volume of cold acetone and then centrifuged at 4 °C for 1 h. The resulting pellet was resuspended in 50 mm phosphate buffer (pH = 7.5) and was subjected to chemical acetylation of the free N terminus amino groups with N‐acetoxy‐[^2^H_3_]‐succinimide.^[^ ^98^ ^]^ After incubating at 30 °C for 90 min, potential *O*‐acetylation of serine, threonine, and tyrosine side chains was reversed by adding 10 µL of 50% hydroxylamine and incubating at room temperature for 20 min. The sample was then cold‐precipitated with acetone to remove chemicals, followed by digestion with trypsin protease for 1 h at 37 °C twice. Peptides were desalted using C18 Spin Columns (Thermo Fisher Scientific) according to the manufacturer's instructions. The retained material was eluted with 80% acetonitrile and 0.1% trifluoroacetic acid followed by evaporation to dryness. Perform strong cation exchange (SCX) of peptides using SCX‐resin containing pipette tips (OMIX SCX‐pipette tips (Agilent)) using the manufacturer's instructions. The enriched peptides were desalted again, dried, and subsequently redissolved for the quantification of N‐terminal peptides by mass spectrometry. For MS analyses, the peptides were resuspended in 10 µL of 0.1% formic acid in 2% acetonitrile and analyzed using an Orbitrap Fusion Lumos (Thermo Fisher Scientific) coupled online to a nano‐LC (Thermo Fisher Scientific) in data‐dependent mode. MS scanning 400–2000 Da at 60 000 resolution using internal calibration. N‐acetylation modified peptides were identified by searching the *Oryza sativa* database using Proteome Discoverer (Thermo Scientific, Ver. 2.4), and significantly altered modification peptides with a *p*‐value <0.05 were validated according to the manufacturer's instructions using a label‐free quantification analysis workflow. Based on the results obtained, proteins showing significant changes with a *q*‐value <0.05 were selected as the final candidates for N‐acetylation modification analysis.

### Determination of Free N Termini

Determination of free N termini was performed as previously described.^[^ ^32^ ^]^ To determine the amount of free N termini with or without *Xoo* inoculation in the wild‐type, *pRESIS*::*RESIS* × *resis*, *resis*, *NAA15*‐RNAi, *NAA10*‐RNAi, soluble proteins were extracted from leaf material (50 mm sodium citrate buffer pH 7.0, and 1 mm EDTA). Protein extracts were subsequently gel‐filtrated via PD SpinTrap G‐25 columns (GE Healthcare) to remove free amino acids. The labeling of free N termini was performed with 2.5 mm extracted protein and 0.5 mm NBD‐Cl in 50 mm sodium citrate buffer (pH 7.0) supplemented with 1 mm EDTA. After 14 h of incubation at 28 °C, the fluorescence intensity was quantified using a Tecan Spark (Tecan; excitation, 470 ± 10 nm; emission, 520 nm).

### FLIM‐FRET Analysis

Protoplasts co‐expressing *pRTV*‐*RESIS*, *pRTV*‐NAA15‐mCherry, and *pRTV‐*NAA10‐eGFP were analyzed by the FLIM‐FRET. Fluorescence confocal imaging and fluorescence lifetime imaging for the analysis were conducted using a scanning confocal microscope SP8 FALCON (Leica Microsystems) with a 100 × objective lens. Picosecond pulsed laser lines (488 nm) from a white light laser system were used as excitation sources. Hybrid photon detectors (HyD1) were used to collect emissions in the ranges of 498–580 nm from the protoplast samples. Fluorescence confocal and lifetime images consisted of 512 × 512 pixels were simultaneously recorded using a galvo‐stage and time‐correlated single‐photon counting technique. All data manipulations were performed and fitted using the Leica suited software (LAS X Ver.3.5.2).

### Polysome Isolation and Fractionation

Total polysome isolation and fractionation were performed as described previously^[^ ^99^, ^100^, ^101^ ^]^ with a little modification. Briefly, 0.2 g leaves were ground in liquid nitrogen and resuspended in 1 mL polysome extraction buffer (0.2 M Tris‐HCl pH 9.0, 0.2 M KCl,0.025 M EGTA, 0.035 M MgCl_2_, 1% Igepal CA630, 5 mm DTT, 1 mm PMSF, 100 µg mL^−1^ cycloheximide, 1% sodium deoxycholate, and 40 U mL^−1^ RNase inhibitor). After 10 min in an ice bath, the lysates were removed by centrifugation at 13 200 g for 15 min at 4 °C. Adjust lysates so that they contain the same OD (260 nm) in 200 µL of lysis buffer. The supernatants were centrifuged for 3 h at 4 °C at 35 000 rpm (SW41Ti rotor in a Beckman L‐100XP ultracentrifuge) in a density gradient of 10% to 50% sucrose. Fractions were collected for each sample with continuous monitoring at 254 nm using a BioComp Piston Gradient Fractionator equipped with a Bio‐Rad Econo UV Monitor. The instrument was calibrated with ultrapure water between samples to ensure accurate readings. The data for each sample were directly compared using the machine‐generated output, without any post‐processing or corrections.

### Bioinformatics Analysis

The RNA‐seq datasets from three rice varieties: *O*. *rufipogon*, *O*. *japonica*, and *O*. *indica* (PRJNA437000^[^ ^36^ ^]^ and PRJNA756899^[^ ^37^ ^]^) were utilized for the identification and conservation analysis of lncRNAs. Raw RNA‐seq data were aligned using HISAT2^[^ ^102^ ^]^ and assembled with StringTie.^[^ ^103^ ^]^ Transcripts with a length ≥ 200 nt and FPKM ≥ 0.1 were retained. Coding potential was assessed using CPC2^[^ ^104^ ^]^ and CNCI^[^ ^105^ ^]^ tools. To identify pathogen‐induced lncRNAs, strand‐specific RNA‐seq was performed on rice leaf samples infected with or without *Xoo*, with three biological replicates. Along with the published dataset (PRJNA544880^[^ ^40^ ^]^ and PRJNA1043097), 74.26% of the reads were used for transcript assembly with Cufflinks, followed by filtering using the Pfam^[^ ^106^ ^]^ and CPC databases. Differential expression analysis was performed using DESeq2 to identify lncRNAs that exhibited significant differential expression under *Xoo* infection. The filter criteria were: log2FoldChange ≥ 2 and *p*‐value <0.05. These lncRNAs were then intersected with the conserved lncRNAs. Nucleic acid and protein sequence homology analyses were both performed using MEGA 11.^[^ ^107^ ^]^ TF prediction and TF binding site prediction were performed using the PlantRegMap.^[^ ^108^ ^]^ ORF prediction was performed using the NCBI ORF Finder, with the following settings: Minimal ORF length (nt) set to 30, Genetic code set to Standard, ORF start codon set to any, and nested ORFs were ignored. EBE elements were predicted using the TAL Effector Nucleotide Targeter 2.0.^[^ ^109^ ^]^

### Statistical Analysis

Statistical analysis used for each dataset can be found in the corresponding figure legend. All plots display means ± SD. The number (n) of biological replicates was at least three. Statistical significance was examined by unpaired, two‐tailed *t*‐tests (^*^ *p* <0.05, ^**^ *p* <0.01, ^***^ *p* <0.001). For multiple comparisons, ANOVA followed by Duncan's new multiple range test was used (*p* <0.05). Statistical analyses were performed using Prism (https://www.graphpad.com/scientific‐software/prism/) and IBM SPSS (https://www.ibm.com/cn‐zh/products/spss). Yield loss was assessed to evaluate the impact of *Xanthomonas oryzae pv. oryzae* (*Xoo*) infection on plant productivity. Grain yield per plant was measured before and after *Xoo* inoculation. yield loss (%) was calculated using the formula: yield loss (%) = [(yield before – yield after) / yield before] × 100%. Figure S2k (Supporting Information) shows the yield before infection, while Figure 2d shows the yield after *Xoo* infection.

## Conflict of Interest

The authors declare no conflict of interest.

## Author Contributions

W.L.Z. conceived and designed the experiments. W.L.Z. and J.H.H. performed the experiments. Y.C. performed the bioinformatics analysis. J.J.F., H.Y.P., and Y.C. analyzed data. Y.C.Q., Z.T.C., and J.P.L. carried out the subcellular localization experiments. Y.F.Z., R.R.H., M.Q.L., and Z.Q.C. carried out the protein purification. L.Y., C.Y., and J.J. carried out the nuclear and cytoplasmic fractionation. Y.Q.C. modified the paper. Y.C.Z. designed the project, wrote the manuscript, and provided funding. The authors read and approved the final manuscript.

## Supporting information



Supporting Information

Supplemental Tables S1‐S9

## Data Availability

The transcriptome sequencing data have been deposited in the NCBI Sequence Read Archive (SRA) under the BioProject accession number (PRJNA1225482). Source data supporting the findings of this study are included in the Supplementary Materials and will be publicly accessible upon publication.
