# Supplementary material for: Gene Editing of a Susceptibility LncRNA Enhances Broad‐Spectrum Disease Resistance in Rice without Developmental Trade‐Offs
Source: Adv Sci (Weinh). 2025 Aug 18;12(47):e05671. doi: 10.1002/advs.202505671 (PMC12713027; doi:10.1002/advs.202505671)
Supplement: Supplementary file 1 — Supporting Information [file ADVS-12-e05671-s001.docx]

**Supplementary information**

**Title: Gene Editing of a Susceptibility LncRNA Enhances Broad-Spectrum Disease Resistance in Rice without Developmental Trade-Offs**

Wen-long Zhao^1^, Ye Cheng^1^, Jia-hui Huang^1^, Jun-jie Feng^1^, Hui-yin Pang^1^, Yi-chao Qin^1^, Zheng-tong Chen^1^, Yu Cheng^1^, Jian-ping Lian^1^, Yan-fei Zhou^1^, Rui-rui He^1^, Meng-qi Lei^1^, Zi-qin Cao^1^, Lu Yang^1^, Chao Yuan^1^, Jie Jiang^1^, Yue-qin Chen^1^, and Yu-chan Zhang^1, *^

**Supplementary Figures 1–7**


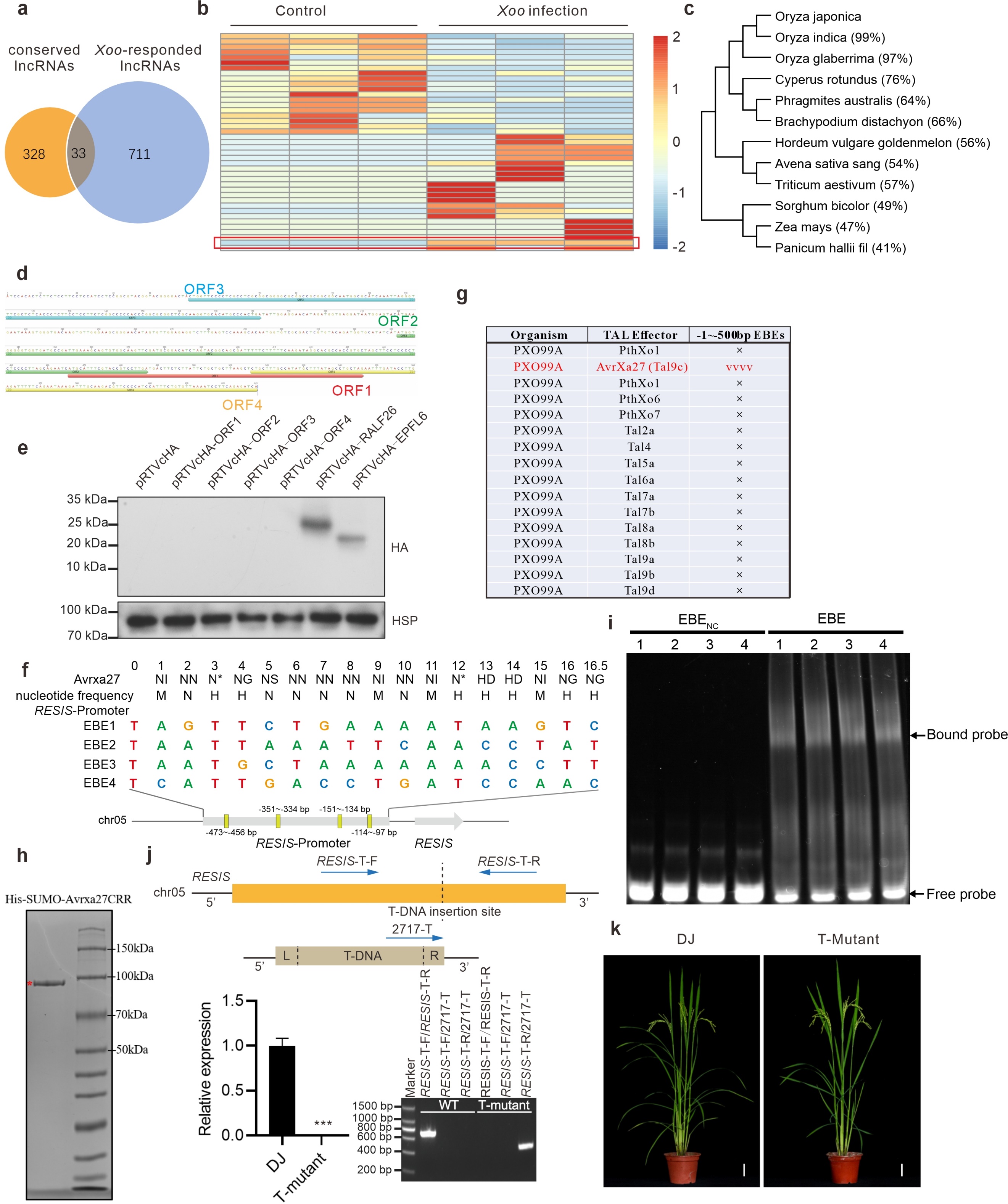


**Figure S1.** Identification of pathogen-induced lncRNAs. Identification of pathogen-induced lncRNAs. **a**, Intersection of conserved and pathogen-responsive lncRNAs. **b**, Expression heatmap of the top 20 lncRNAs with significant expression changes under *Xoo* infection. The red box highlights *RESIS*. **c**, phylogenetic analysis of *RESIS* in monocots. The percentage represents the sequence alignment rate. **d**, Schematic diagram of the ORFs prediction results for the *RESIS* transcript. e, Western blot validation results of the four predicted ORFs. RALF26 and EPFL6 were used as small peptide controls. **f**, Schematic representation of the nucleotide sequence of the *RESIS* EBE elements and the corresponding nucleotide sequence recognized by the RVDs of AvrXa27. Nucleotide frequency shows the most common degenerate bases recognized by AvrXa27 (M: A/C, H: A/C/T, N: A/ G/C/T). **g**, TAL effector prediction on *RESIS* promoter region. **h**, Coomassie Brilliant Blue staining of recombinant purified Avrxa27CRR proteins with the indicated His-SUMO tags for EMSA assay. The red asterisk indicates the correct band. **i**, EMSA showing the binding of His-tagged AvrXa27CRR to *RESIS* EBEs and the control probe EBE_NC_. **j**, Evaluation of the effects of the *RESIS* T-DNA mutant. **k**, The whole plants of the wild-type control DJ and the T-mutant of *RESIS* at heading stage. Scale bars, 5 cm. Statistics: n = 3 per group for **e**. Data shown represent mean ± SD. Unpaired, two-tailed t-tests were performed. ***P < 0.001.


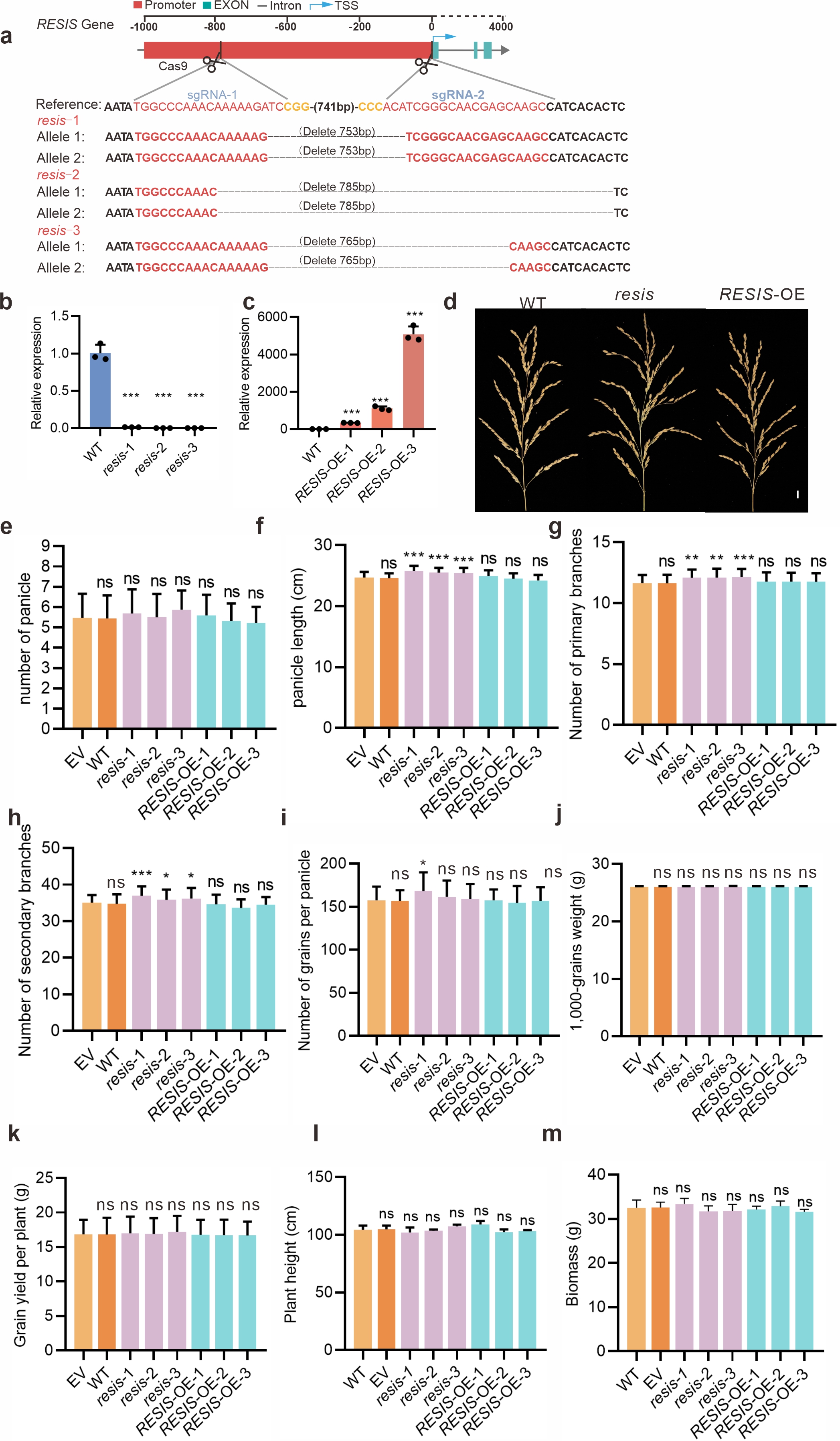


**Figure S2.** Construction and phenotypic analysis of *RESIS*-OE and *resis* transgenic plants. **a**, The CRISPR/Cas9 editing sites of two targets on *RESIS*. The protospacer adjacent motif (PAM) is shown in red. **b**, Relative expression levels of *RESIS* in *resis* transgenic lines. **c**, Relative expression levels of *RESIS* in *RESIS*-OE transgenic lines. **d**, Representative panicles of WT, *resis* and *RESIS*-OE plants. Scale bar, 1 cm. **e**–**m**, Statistically analysis of the panicle number (**e**), panicle length (**f**), number of primary branches per panicle (**g**), number of secondary branches per panicle (**h**), number of grains per panicle (**i**),1,000-grains weight (**j**), grain yield per plant (**k**), plant height (**l**) and biomass (**m**) in EV (empty vector), WT and transgenic plants. Statistics: n = 3 per group for **b** and **c**; n ≥ 30 per group for **e**–**k**; and n ≥ 3 per group for **l** and **m**. Data shown represent mean ± SD. Unpaired, two-tailed *t*-tests were performed. **P* < 0.05, ***P* < 0.01 and ****P* < 0.001.


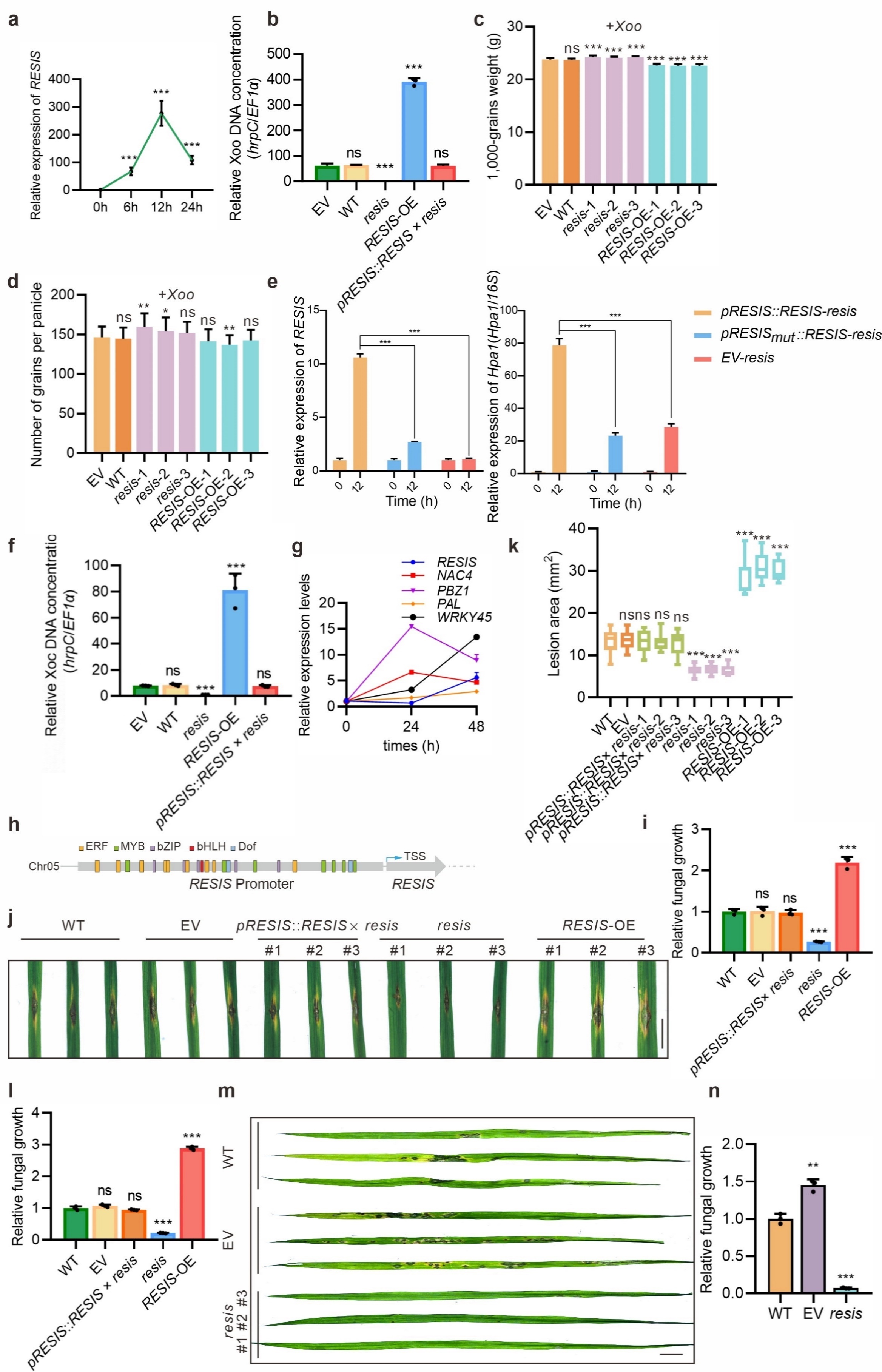


**Figure S3.** Pathogen resistance analysis of EV, WT, *resis*, *RESIS*-OE and *pRESIS*::*RESIS* × *resis* plants*.* **a**, Relative expression levels of *RESIS* in WT leaves after *Xoo* inoculation by qPCR analysis. Normalized relative to the time point 0. **b**, qPCR analysis of relative *Xoo* DNA concentration between bacterial *hrpC* and rice *EF1α* in EV (empty vector), WT, *resis*, *RESIS*-OE and *pRESIS*::*RESIS* × *resis*. **c**, Statistically analysis of the 1,000-grains weight after inoculation with *Xoo* strains PXO99A. **d**, Statistically analysis of the number of grains per panicle after inoculation with *Xoo* strains PXO99A. **e**, *RESIS* expression and *Xoo* infection assessment after transferring *pRESIS*::*RESIS* vector, an empty vector (EV), and a *pRESISmut*::*RESIS* vector (in which the EBEs were mutated) into *resis* protoplasts with *Xoo*. **f**, qPCR analysis of relative *Xoc* DNA concentration between bacterial *hrpC* and rice *EF1α* in EV, WT, *resis*, *RESIS*-OE and *pRESIS*::*RESIS* × *resis*. **g**, Relative expression levels of *RESIS* blast-responsive genes in WT leaves after *M*. *oryzae* inoculation by qPCR analysis. Normalized relative to the time point 0. **h**, Analysis of upstream TF binding elements in *RESIS* promoter. **i**, Relative fungal growth of *M*. *oryzae* strain 08-T19 in WT, EV, *pRESIS*::*RESIS* × *resis*, *resis* and *RESIS*-OE plant leaves at tillering stage. **j**, Phenotypes of the seedling leaves of WT, EV, *pRESIS*::*RESIS* × *resis*, *resis* and *RESIS*-OE plants punch-inoculated with the *M*. *oryzae* strain 08-T19. Scale bar, 1 cm. **k**, The lesion area of (**j**) was measured at 7 dpi. **l**, Relative fungal growth of *M*. *oryzae* strain 08-T19 in WT, EV, *pRESIS*::*RESIS* × *resis*, *resis* and *RESIS*-OE seedling leaves. **m**, Phenotypes of the seedling leaves of WT, EV and *resis* plants sprayed with the *M*. *oryzae* strain 08-T19. Scale bar, 1 cm. **n**, Relative fungal growth of *M*. *oryzae* strain 08-T19 in WT, EV and *resis* plants at 7 dpi. Statistics: n = 3 per group for **a**, **b**, **e**, **f**, **g, i, l** and **n**; n = 30 per group for **c** and **d**; and n ≥ 6 per group for **k**. Data shown represent mean ± SD. Unpaired, two-tailed *t*-tests were performed. **P* < 0.05, ***P* < 0.01 and ****P* < 0.001.


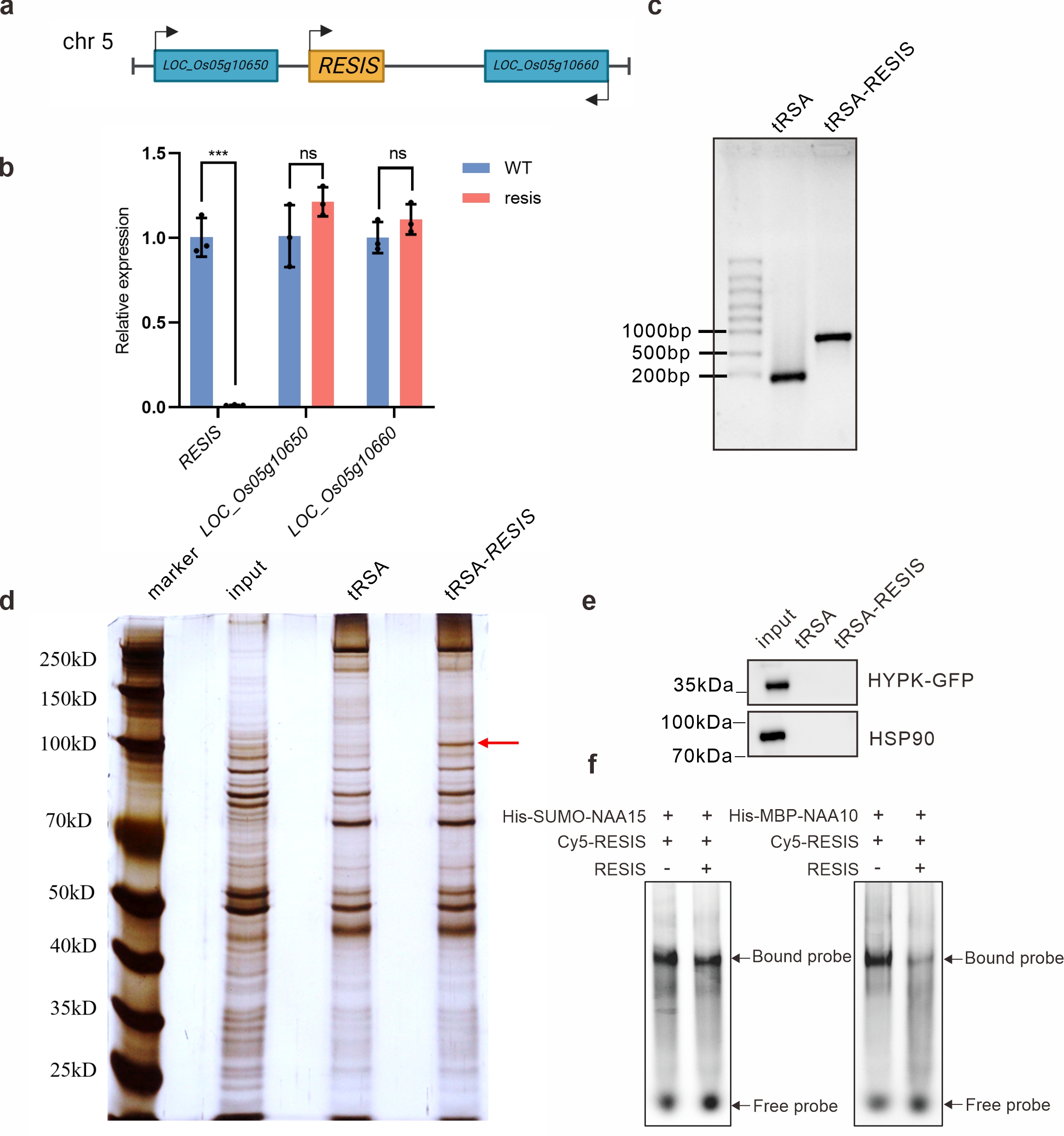


**Figure S4.** Mechanism analysis of *RESIS*. **a**, Diagram of the *RESIS* locus and its neighboring genes. The arrows indicate transcriptional direction. **b**, Relative expressions of the neighboring genes of *RESIS* in WT and T-mutant. **c**, Diagram of formaldehyde denaturing gel showed *in vitro* transcription RNA of tRSA and tRSA*-RESIS*. **d**, RNA pull-down assay of *RESIS*. tRSA was used as negative control. **e**, RNA pull-down assay of *RESIS* with HYPK. **f**, RNA EMSA showing *RESIS* binding to NAA15 and NAA10. The red arrow indicates the interacted protein of *RESIS*. Statistics: n = 3 per group for **b**. Data shown represent mean ± SD. Unpaired, two-tailed t-tests were performed. ***P < 0.001.


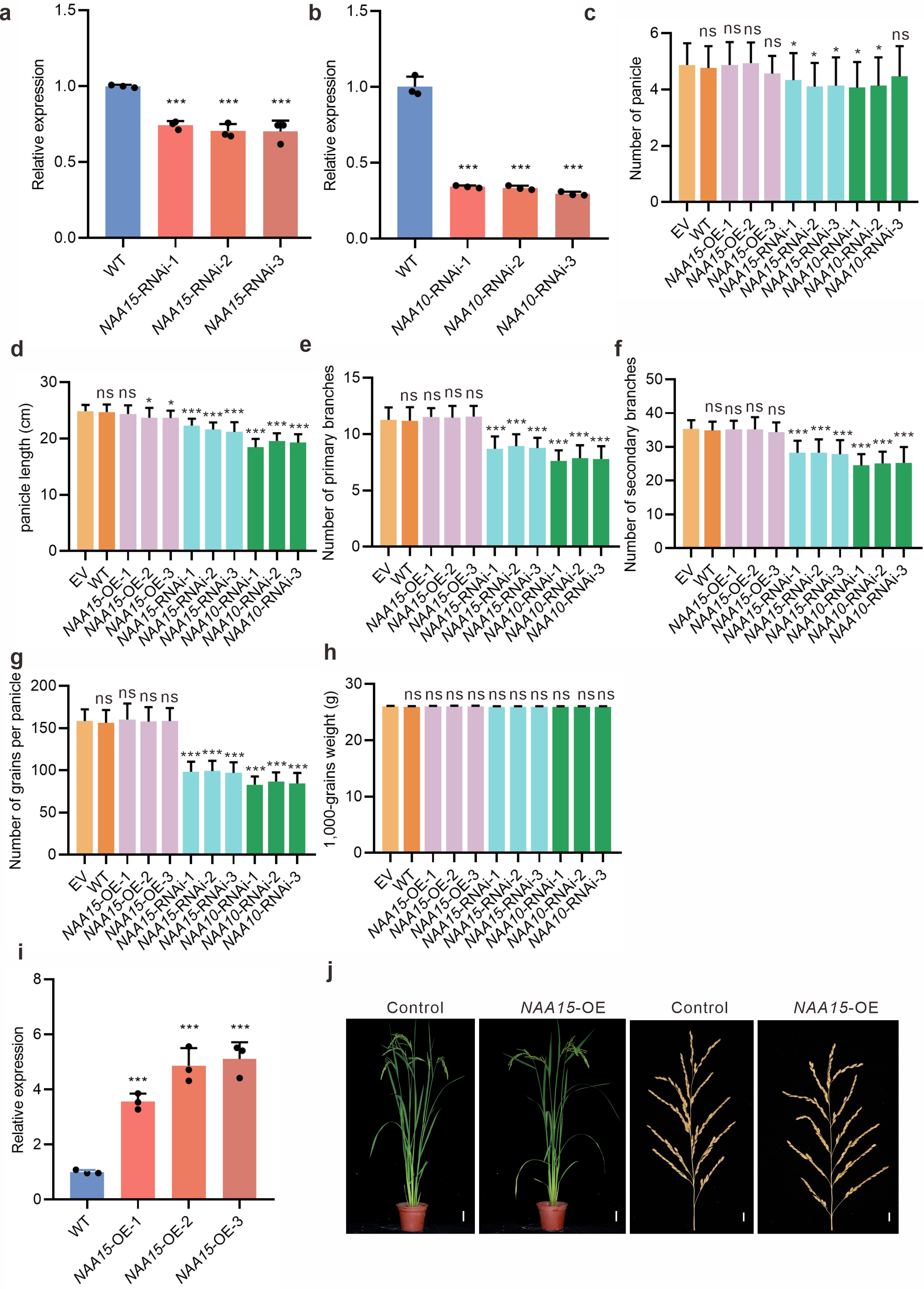


**Figure S5.** Phenotypical analysis of *NAA15*-RNAi, *NAA15*-OE, and *NAA10*-RNAi transgenic plants. **a**, Relative expressions of *NAA15*-RNAi transgenic lines. b, Relative expressions of *NAA10*-RNAi transgenic lines. **c**–**h**, Statistically analysis of the panicle number (**c**), panicle length (**d**), number of primary branches per panicle (**e**), number of secondary branches per panicle (**f**), number of grains per panicle (**g**), and1,000-grains weight (**h**) in EV, WT and transgenic plants. **i**, Relative expressions of *NAA15*-OE transgenic lines. **j**, The whole plants and the representative panicles of wild-type control and the *NAA15*-OE at heading stage. Scale bars, 5 cm for whole plants, and 1 cm for panicles. Statistics: n = 3 per group for **a**–**c**; and n = 30 per group for **e**–**j**. Data shown represent mean ± SD. Unpaired, two-tailed *t*-tests were performed. **P* < 0.05 and ****P* < 0.001.


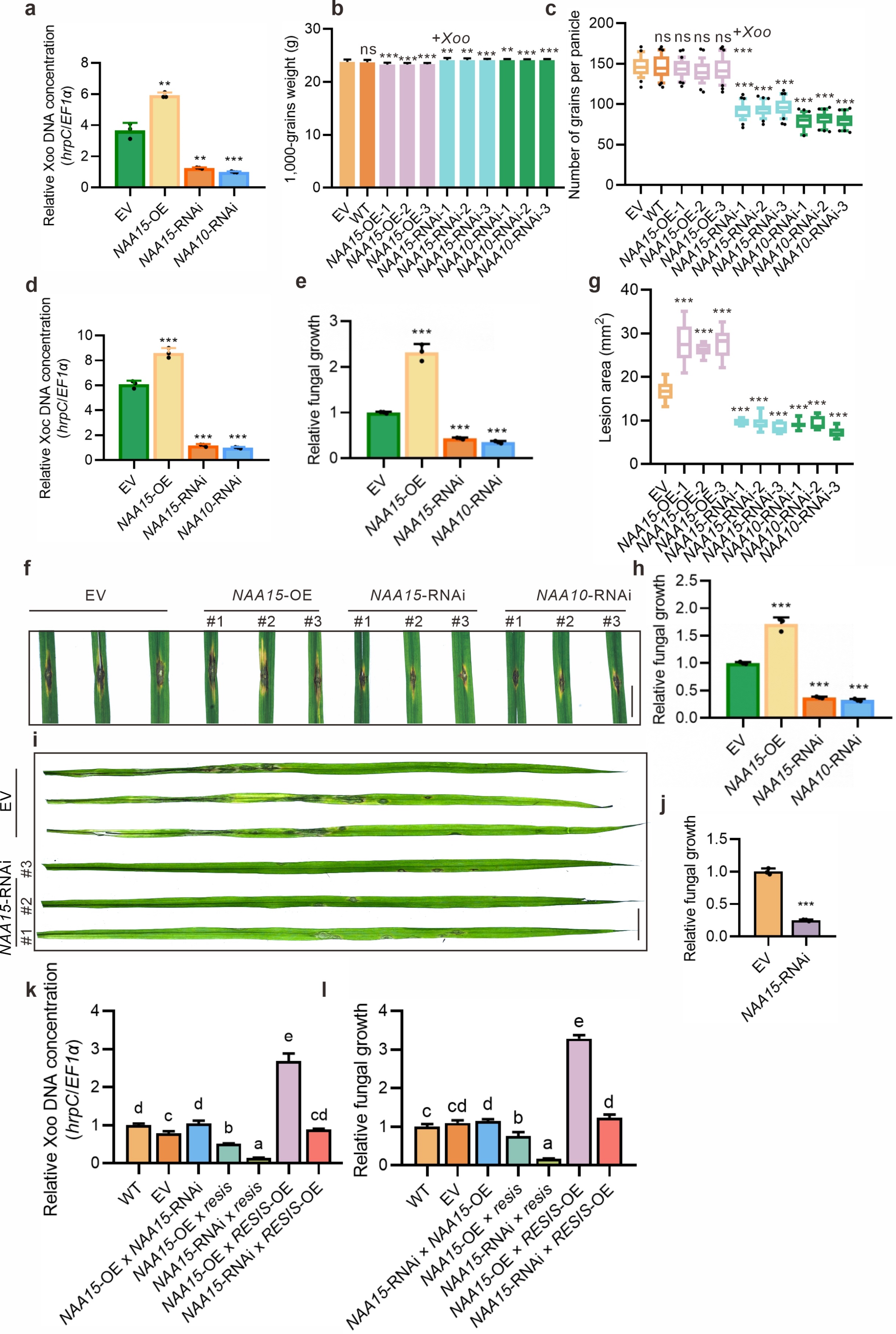


**Figure S6.** Pathogen resistance analysis of control, *NAA15*-OE, *NAA15*-RNAi and *NAA10*-RNAi plants*.* **a**, qPCR analysis of relative *Xoo* DNA concentration between bacterial *hrpC* and rice *EF1α* in EV, *NAA15*-OE, *NAA15*-RNAi, *NAA10*-RNAi. **b**, 1,000-grains weight of EV, WT, *NAA15*-OE, *NAA15*-RNAi and *NAA10*-RNAi after inoculation *Xoo*. **c**, Number of grains per panicle of EV, WT, *NAA15*-OE, *NAA15*-RNAi and *NAA10*-RNAi after inoculation *Xoo*. **d**, qPCR analysis of relative *Xoc* DNA concentration between bacterial *hrpC* and rice *EF1α* in EV, *NAA15*-OE, *NAA15*-RNAi, *NAA10*-RNAi. **e**, Relative fungal growth of *M*. *oryzae* strain 08-T19 in EV, *NAA15*-OE, *NAA15*-RNAi and *NAA10*-RNAi plant leaves at tillering stage. **f**, Phenotypes of the seedling leaves of EV and *NAA15*-RNAi plants punch-inoculated with the *M*. *oryzae* strain 08-T19. Scale bar, 1 cm. **g**, The lesion area of (**f**) was measured at 7 dpi. **h**, Relative fungal growth of *M*. *oryzae* strain 08-T19 in EV, *NAA15*-OE*, NAA15*-RNAi and *NAA10*-RNAi seedling leaves. **i**, Phenotypes of the seedling leaves of EV and *NAA15*-RNAi plants sprayed with the *M*. *oryzae* strain 08-T19. Scale bar, 1 cm. **j**. Relative fungal growth of *M*. *oryzae* strain 08-T19 in WT, EV and *resis* plants at 7 dpi. **k**, qPCR analysis of relative *Xoo* DNA concentration between bacterial *hrpC* and rice *EF1α* in genetic complementation plants. **l**, Relative fungal growth of *M*. *oryzae* strain 08-T19 in genetic complementation plants. Statistics: n = 3 per group for **a**, **d**, **e**, **h**, **j**, **k** and **l**; n = 30 per group for **b** and **c**; and n ≥ 6 per group for **g**. Data shown represent mean ± SD. Unpaired, two-tailed *t*-tests were performed in **a**–**e** and **g**, **h**, **j**. ***P* < 0.01 and ****P* < 0.001. one-way ANOVA with Duncan’s new multiple range test in **k** and **l**. Significant differences are indicated by different letters.


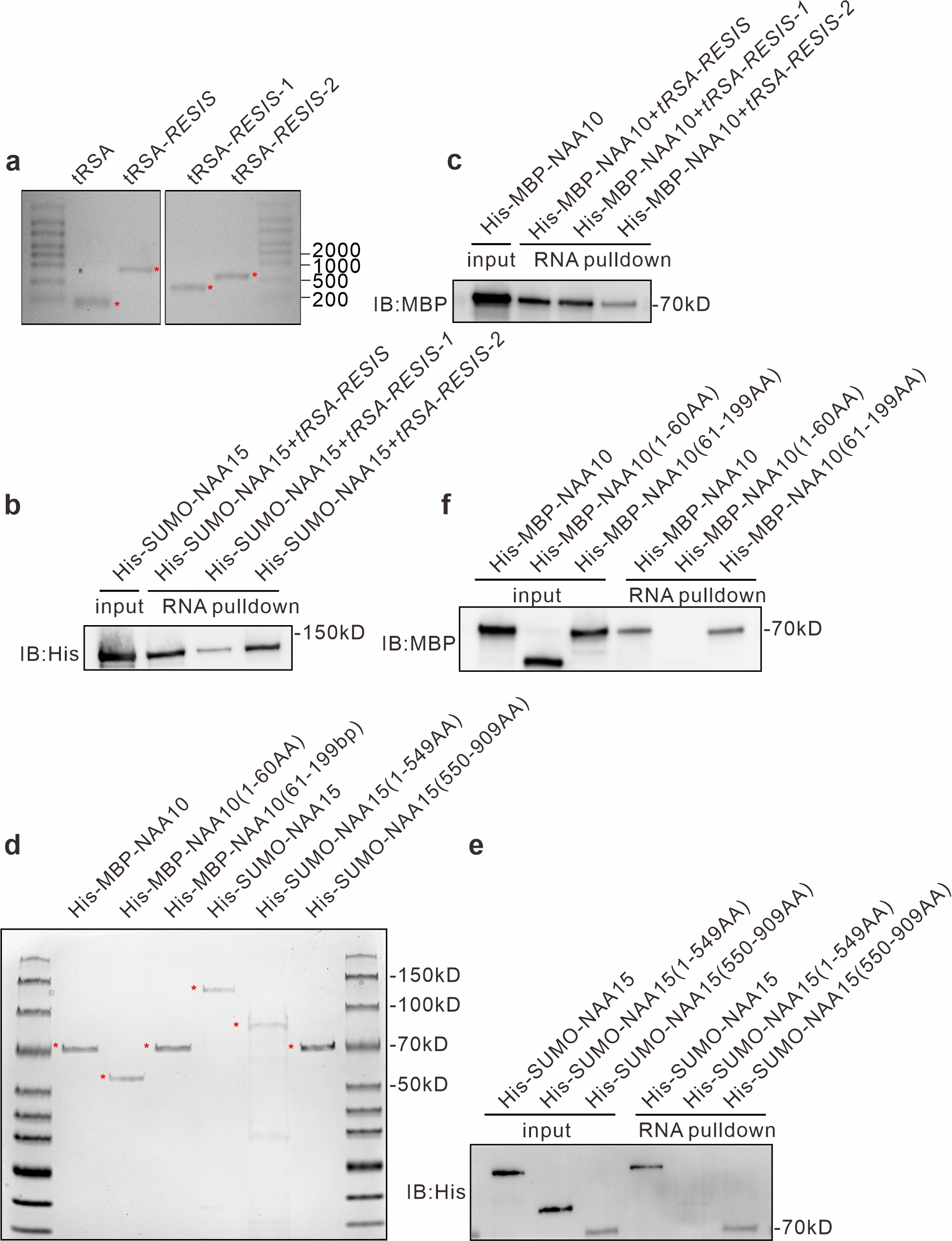


**Figure S7.** Regulation of the NatA complex by *RESIS*. **a**, Diagram of formaldehyde denaturing gel showed *in vitro* transcription RNA of tRSA, tRSA*-RESIS*, tRSA*-RESIS*-1 and tRSA*-RESIS*-2. The red asterisks indicate the correct band. **b**, RNA pull-down assay of NAA15 by different *RESIS* fragments. The *RESIS* transcript was divided into two fragments: 1 (1–214 nt) and 2 (215–629 nt). The full-length of NAA15 was purified from recombinant His-SUMO protein. The full-length and the two fragments of *RESIS* were fused to tRSA. **c**, RNA pull-down assay of NAA10 by different *RESIS* fragments. The *RESIS* transcript was divided into two fragments: 1 (1–214 nt) and 2 (215–629 nt). The full-length of NAA10 was purified from recombinant His-MBP protein. The full-length and the two fragments of *RESIS* were fused to tRSA. **d**, Coomassie Brilliant Blue staining of recombinant purified NAA10, NAA10 (1–60 AA), NAA10 (61–199 AA), NAA15, NAA15 (1–549 AA) and NAA15 (550–909 AA) proteins with the indicated His-MBP or His SUMO tags for *in vitro* assay. The red asterisks indicate the correct band. **e**, Identification of the NAA15-binding domain on *RESIS* by RNA pull-down and western blot. The full-length and two fragments of NAA15 was purified from recombinant His-SUMO protein. The full-length of *RESIS* were fused to tRSA. **f**, Identification of the NAA10-binding domain on *RESIS* by RNA pull-down and western blot. The full-length and two fragments of NAA10 was purified from recombinant His-MBP protein. The full-length of *RESIS* were fused to tRSA.


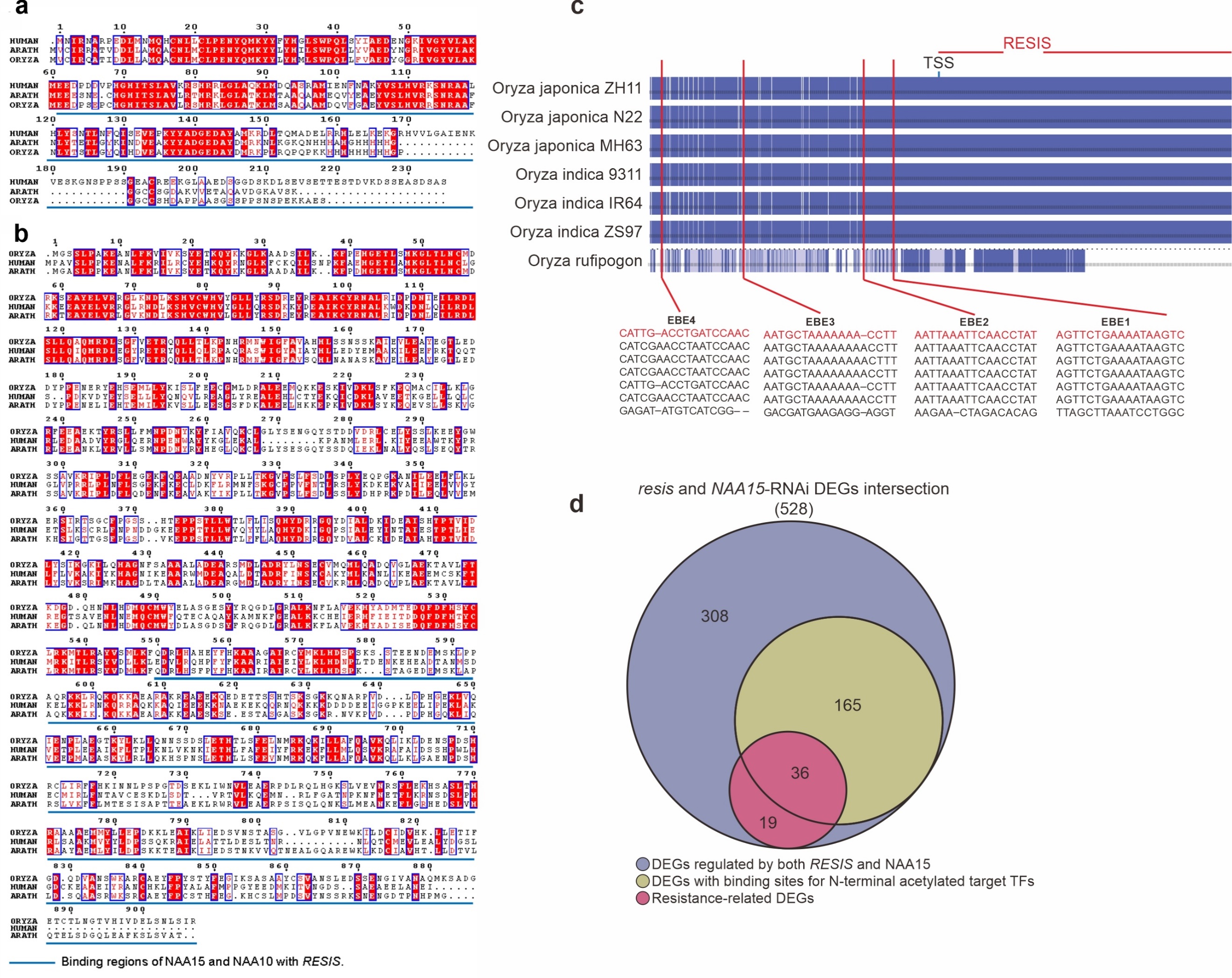


**Figure S8.** Conservation analysis of NAA10, NAA15 and *RESIS*. **a** and **b**, Homology comparison of NAA10 (**a**) and NAA15 (**b**) in *Oryza sativa*, *Arabidopsis thaliana*, and *Homo sapiens*. **c**, EBEs in *RESIS* promoters across six cultivars and one wild rice. **d**, Schematic diagram of the proportion of the DEGs.
